# Deletion of *nuoG* from the Vaccine Candidate *Mycobacterium bovis* BCG Δ*ureC*::*hly* Improves Protection against Tuberculosis

**DOI:** 10.1128/mBio.00679-16

**Published:** 2016-05-24

**Authors:** Martin Gengenbacher, Natalie Nieuwenhuizen, Alexis Vogelzang, Haipeng Liu, Peggy Kaiser, Stefanie Schuerer, Doris Lazar, Ina Wagner, Hans-Joachim Mollenkopf, Stefan H. E. Kaufmann

**Affiliations:** a Department of Immunology, Max Planck Institute for Infection Biology, Berlin, Germany; b Department of Microbiology and Immunology, National University of Singapore, Yong Loo Lin School of Medicine, Singapore; c Max Planck Institute for Infection Biology, Core Facility Genomics/Microarray, Berlin, Germany

## Abstract

The current tuberculosis (TB) vaccine, *Mycobacterium bovis* Bacillus Calmette-Guérin (BCG), provides insufficient protection against pulmonary TB. Previously, we generated a listeriolysin-expressing recombinant BCG strain, which to date has successfully completed phase I and phase IIa clinical trials. In an attempt to further improve efficacy, we deleted the antiapoptotic virulence gene *nuoG*, encoding NADH dehydrogenase 1 subunit G, from BCG Δ*ureC*::*hly*. *In vitro*, deletion of *nuoG* unexpectedly led to strongly increased recruitment of the autophagosome marker LC3 to the engulfed vaccine, suggesting that *nuoG* also affects xenophagic pathways. In mice, BCG Δ*ureC*::*hly* Δ*nuoG* vaccination was safer than BCG and improved protection over that of parental BCG Δ*ureC*::*hly*, significantly reducing TB load in murine lungs, ameliorating pulmonary pathology, and enhancing immune responses. Transcriptome analysis of draining lymph nodes after vaccination with either BCG Δ*ureC*::*hly* or BCG Δ*ureC*::*hly* Δ*nuoG* demonstrated earlier and stronger induction of immune responses than that with BCG SSI and suggested upregulation of inflammasome activation and interferon-induced GTPases. In summary, BCG Δ*ureC*::*hly* Δ*nuoG* is a promising next-generation TB vaccine candidate with excellent efficacy and safety.

## IMPORTANCE

Autophagy and apoptosis are fundamental processes allowing cells to degrade their components or kill themselves, respectively. The immune system has adopted these mechanisms to eliminate intracellular pathogens. Residing in host cells, the causative agent of TB, *Mycobacterium tuberculosis*, has evolved strategies to set cellular programs of autophagy and apoptosis “on hold.” The mycobacterial gene *nuoG* was found to prevent host cell apoptosis. We have deleted *nuoG* in the live vaccine candidate BCG Δ*ureC*::*hly*, which is in phase II clinical development, to leave cellular apoptosis “on go” upon immunization. In preclinical models, this strategy boosted immunity and improved protection from *M. tuberculosis* infection. Unexpectedly, we obtained compelling evidence that mycobacterial *nuoG* facilitates inhibition of autophagic pathways, suggesting a new role for this gene in the host-pathogen interplay in TB.

## INTRODUCTION

Tuberculosis (TB) remains a global health burden (1). The TB vaccine *Mycobacterium bovis* bacillus Calmette-Guérin (BCG), which was attenuated by serial passage of the pathogen *M. bovis*, entered clinical practice in 1924 (2). BCG has contributed to reduction of childhood mortality and is relatively safe in immunocompetent individuals. However, it fails to prevent the most prevalent form of disease, pulmonary TB, and disease transmission and carries an increased risk of adverse events in HIV^+^ infants (3, 4). Previously, we developed a recombinant derivative, BCG Δ*ureC*::*hly*, with significantly improved protective efficacy and safety in mice by secretion of listeriolysin O, a pore-forming protein of *Listeria monocytogenes* (5–7). The vaccine has successfully completed phase I and IIa clinical trials (NCT01479972, NCT01113281, and NCT00749034), demonstrating its safety and immunogenicity in adults and infants (8, 9). *In vitro*, BCG *ΔureC*::*hly* induced increased apoptosis, inflammasome activation, and expression of microtubule-associated protein light chain 3 (LC3) (7, 8). In mice, BCG *ΔureC*::*hly* induced increased central memory CD4^+^ T cells with protective capacity and expression of *IL-1β*, *IL-18*, and *Tmem173* (*STING*) (6, 7).

Immune cells have adopted crucial processes, such as programmed cell death (apoptosis) and intracellular degradation of host cell compartments (autophagy), for antimicrobial defense (10). Selective autophagy, also termed xenophagy, in which cells direct autophagic pathways against pathogens, is a mechanism of intracellular killing of *Mycobacterium tuberculosis* (11–13) and also promotes antigen presentation (14). After *M. tuberculosis* infection, bacterial DNA and proteins translocate into the host cell cytosol by the *Mycobacterium*-specific type VII secretion system, Esx-1, and they are subsequently recognized by autophagy receptors p62 and Ndp51 (11–13). BCG lacks Esx-1, does not rupture the phagosome, and is not targeted by autophagy under normal conditions (7, 11, 14, 15). Apoptosis controls *M. tuberculosis* replication and spreading (16), while phagocytosis of apoptotic host cell-derived vesicles by dendritic cells boosts T cell responses via cross-presentation (17). *M. tuberculosis* has evolved strategies to inhibit these defense mechanisms. Screening for antiapoptotic genes in *M. tuberculosis* identified *nuoG*, encoding a subunit of the dispensable respiratory enzyme complex NADH dehydrogenase 1 (18, 19). Disruption of *nuoG* increased *M. tuberculosis*-induced apoptosis via a tumor necrosis factor alpha (TNF-α)-dependent mechanism and decreased the virulence of *M. tuberculosis* (18). As apoptosis is thought to enhance adaptive immune responses through cross-presentation (17, 20, 21), we deleted *nuoG* in BCG *ΔureC*::*hly.* We were able to surmount the high bar, further increasing efficacy against TB in a vaccine that already expresses 100-fold-higher protection than BCG, without loss of its excellent safety profile*.*

## RESULTS

### Disruption of *nuoG* in BCG and BCG Δ*ureC*::*hly* induces colocalization of bacteria with the autophagosomal marker LC3.

**FIG 1:**
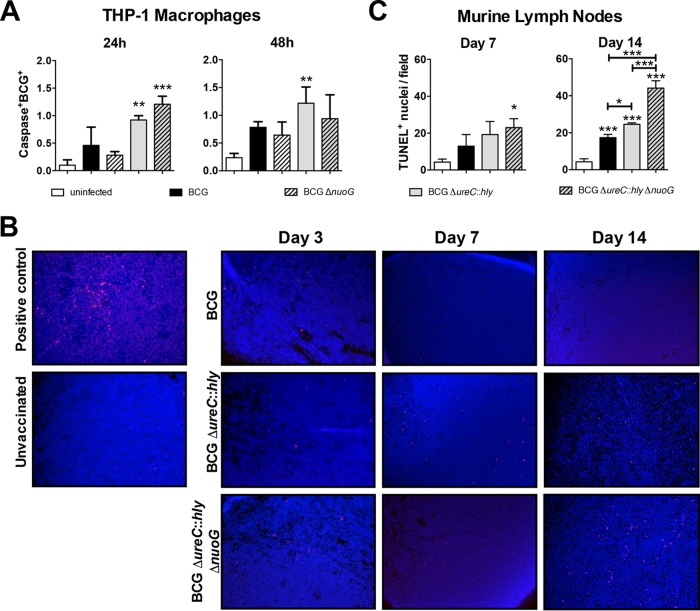
Effect of mycobacterial *nuoG* deletion on apoptosis of host THP-1 macrophages and in lymph nodes of vaccinated mice. (A) Percentages of caspase-3/7^+^ BCG^+^ THP-1 macrophages were quantified in triplicate samples at 24 h and 48 h by ArrayScan after infection with PKH26-labeled BCG, BCG Δ*nuoG*, BCG Δ*ureC*::*hly*, and BCG Δ*ureC*::*hly* Δ*nuoG* (MOI of 20) for 4 h. Results are representative of three experiments. (B) TUNEL staining of lymph node sections at days 3, 7, and 14 postvaccination. A lymph node from an *M. tuberculosis*-infected mouse was used as a positive control. Purple spots are TUNEL^+^ (apoptotic cells). Cell nuclei were stained with 4′,6-diamidino-2-phenylindole. (C) Quantification of apoptotic cells in lymph nodes at days 7 and 14 postvaccination. TUNEL^+^ cells per field of view at 20× (*n* = 3 mice; both dLNs) were counted. Results are representative of two experiments. Data were analyzed using one-way ANOVA with Tukey’s multiple-comparison test. *, *P* < 0.05; **, *P* < 0.01; ***, *P* < 0.001.

We deleted *nuoG* from BCG Δ*ureC*::*hly* in an attempt to further improve vaccine efficacy by enhancing apoptosis. Previously, Velmurugan et al. showed increased apoptosis in THP-1 macrophages following deletion of the *nuoG* gene from *M. tuberculosis* (19). Here, disruption of *nuoG* in BCG and BCG Δ*ureC*::*hly* did not increase apoptosis of infected THP-1 macrophages at 24 h or 48 h postinfection (p.i.), although both listeriolysin-expressing strains, BCG Δ*ureC*::*hly* and its *nuoG*-deficient derivative, tended to induce more apoptosis than BCG (Fig. 1A). After vaccination of mice with BCG Δ*ureC*::*hly* and BCG Δ*ureC*::*hly* Δ*nuoG*, numbers of apoptotic cells remained unchanged in draining lymph nodes (dLNs) compared to BCG-vaccinated mice at early time points (Fig. 1B and C). However, by day 14, both BCG Δ*ureC*::*hly* and BCG Δ*ureC*::*hly* Δ*nuoG* significantly increased apoptosis in comparison to BCG, which was further enhanced in the absence of *nuoG*, suggesting a downstream effect on apoptosis (Fig. 1B and C).

**FIG 2:**
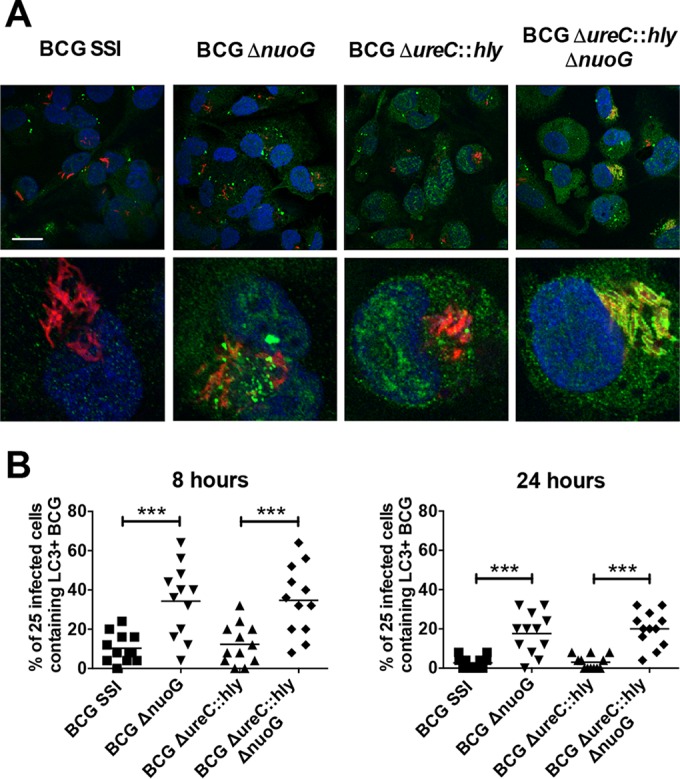
Deletion of *nuoG* from BCG and BCG Δ*ureC*::*hly* leads to increased association of LC3 with engulfed bacteria in THP-1 macrophages. (A) LC3 staining (green) in THP-1 macrophages infected with PKH26-labeled BCG SSI, BCG Δ*nuoG*, BCG Δ*ureC*::*hly*, and BCG Δ*ureC*::*hly* Δ*nuoG* (red), 24 h p.i. Nuclei are stained with Hoechst stain (blue). Results are representative of three experiments. (Top) Bar, 10 µm; (bottom) cropped images showing a single cell. (B) Autophagy quantification in THP-1 macrophages at 8 and 24 h p.i. Individual points represent the percentages of 25 infected cells containing LC3-associated bacteria, and 300 cells were counted per group. Two experiments. Data were analyzed using one-way ANOVA with Tukey’s multiple-comparison test. ***, *P* < 0.001.

Further experiments in THP-1 macrophages demonstrated that, unexpectedly, knockout of *nuoG* from BCG strains drastically enhanced colocalization of the vaccine with the autophagy protein LC3 (Fig. 2A), from 4 to 8 h p.i. up to 48 h p.i. (Fig. 2A and B). While LC3 was previously shown to be increased in THP-1 macrophages after BCG Δ*ureC*::*hly* infection (7), it did not specifically colocalize with the vaccine as seen after infection with BCG Δ*ureC*::*hly* Δ*nuoG*. This suggests an intriguing new role for the mycobacterial gene *nuoG* in suppressing host cell xenophagic responses, which may involve either the canonical autophagy pathway or LC3-associated phagocytosis (LAP), two mechanistically distinct processes involving autophagy proteins (22, 23).

### Deletion of *nuoG* improves vaccine-induced protection.

**FIG 3:**
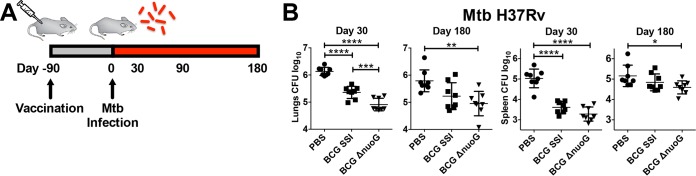
Deletion of *nuoG* in BCG improves protection of mice against TB. (A) Schematic design of protection studies. (B) Mice vaccinated subcutaneously with phosphate-buffered saline (PBS; ●), BCG SSI (■), or BCG Δ*nuoG* (▼) were aerosol infected after 90 days with a low dose of 100 to 200 CFU of *M. tuberculosis* laboratory strain H37Rv. Bacterial burdens of lungs and spleen were determined at 30 and 180 days postchallenge by plating organ homogenates on agar. Shown are means ± standard deviations. Results are representative of two experiments. Data were analyzed using one-way ANOVA with Tukey’s multiple-comparison test (*n* = 8). *, *P* < 0.05; **, *P* < 0.01; ***, *P* < 0.001; ****, *P* < 0.0001.

**FIG 4:**
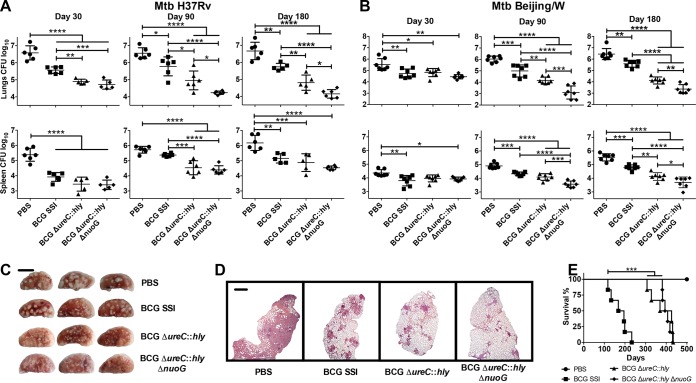
Deletion of *nuoG* in the clinical vaccine candidate, BCG Δ*ureC*::*hly*, further improves long-term protection against TB. (A and B) Mice vaccinated subcutaneously with phosphate-buffered saline (PBS; ●), BCG SSI (■), BCG Δ*ureC*::*hly* (▲), or BCG Δ*ureC*::*hly* Δ*nuoG* (◆) were aerosol infected after 90 days with a low dose of 100 to 200 CFU of *M. tuberculosis* laboratory strain H37Rv (*n* = 5 to 6) per animal (A) or a clinical isolate of the Beijing/W lineage (*n* = 7) (B). Bacterial burdens of organs were assessed at designated time points p.i. by plating organ homogenates. Shown are means ± standard deviations. Results are representative of three (A) or two (B) experiments. Data were analyzed using one-way ANOVA with Tukey’s multiple-comparison test. (C) Gross pathology of left lung lobe from three out of six vaccinated mice 180 days p.i. with *M. tuberculosis* H37Rv. Bar, 5 mm. Results are representative of two experiments. (D) Pulmonary histopathology of the left lung lobe from vaccinated mice 180 days p.i. with *M. tuberculosis* H37Rv. Lungs were fixed and embedded, and sections were stained with H&E prior to examination. Bar, 1 mm. Results are representative of two experiments. (E) Survival of SCID mice after subcutaneous administration of 10^6^ CFU of indicated strains was monitored over time. Median survival calculated by Mantel-Cox log rank test was 181.5 days (BCG SSI), 398 days (BCG Δ*ureC*::*hly*), and 405 days (BCG Δ*ureC*::*hly* Δ*nuoG*). Results are representative of two experiments. *, *P* < 0.05; **, *P* < 0.01; ***, *P* < 0.001; ****, *P* < 0.0001.

To assess the specific influence of *nuoG* deletion on vaccine efficacy, we immunized mice with BCG Δ*nuoG* and determined bacterial loads over 180 days post-*M. tuberculosis* challenge (Fig. 3A). Vaccination with BCG Δ*nuoG* consistently reduced the *M. tuberculosis* burden in lungs of mice over that after vaccination of mice with BCG SSI, with a similar, less pronounced trend in spleens (Fig. 3B). Having demonstrated that *nuoG* disruption improved protective efficacy, we then investigated whether this effect synergizes with the apoptosis-inducing phenotype of BCG Δ*ureC*::*hly*. Consistent with previous reports, BCG Δ*ureC*::*hly*-vaccinated mice were better protected than BCG SSI-vaccinated mice (Fig. 4A and B) (5, 24). Importantly, additional deletion of *nuoG* further improved efficacy against challenge with both an *M. tuberculosis* laboratory strain (H37Rv) (Fig. 4A) and a clinical *M. tuberculosis* isolate (Beijing/W lineage) (Fig. 4B). Protection was particularly improved in lungs, which also benefited from markedly ameliorated gross pathology (Fig. 4C) and histopathology (Fig. 4D) at 180 days p.i. Differences in bacterial counts were less pronounced but statistically significant in spleens (Fig. 4A and B). Thus, BCG Δ*ureC*::*hly* Δ*nuoG* conferred increased protection compared not only to BCG but also to BCG Δ*ureC*::*hly*, against both pulmonary and disseminated TB. In order to assess the safety of the recombinant BCG vaccine candidates, we examined persistence and dissemination in the months following vaccination. The two recombinant vaccine candidates were comparable, i.e., more quickly cleared from the lymph nodes than BCG SSI and with lower degrees of dissemination to the spleen and no dissemination to the lung (see Fig. S1 in the supplemental material). Attenuation of the recombinant strains compared to BCG SSI was confirmed by studies in severe combined immunodeficiency (SCID) mice, in which BCG Δ*ureC*::*hly*- and BCG Δ*ureC*::*hly* Δ*nuoG*-vaccinated mice survived twice as long as BCG SSI-vaccinated animals. Together, our data demonstrate that deletion of *nuoG* sustained the notable preclinical safety profile of BCG Δ*ureC*::*hly* (Fig. 4E) (5). In summary, deletion of *nuoG* from BCG Δ*ureC*::*hly* improved efficacy against TB, paralleled by excellent safety.

### Vaccination with BCG Δ*ureC*::*hly* Δ*nuoG* enhanced immune responses.

**FIG 5:**
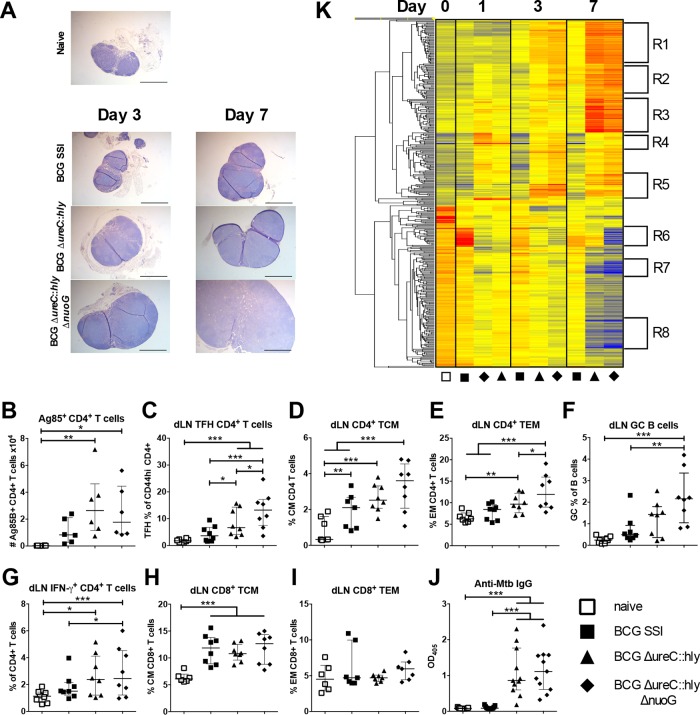
Enhanced immune responses after vaccination with BCG Δ*ureC*::*hly* and BCG Δ*ureC*::*hly* Δ*nuoG* compared to BCG SSI. (A) Lymph nodes stained with H&E (*n* = 4 or 5, two experiments). Bar, 1 mm. (B) Frequencies of Ag85B^+^ CD4^+^ T cells at day 14 postvaccination. (C to F) Frequencies of T follicular helper (TFH) cells (C), CD4^+^ central memory T (TCM) cells (D), effector memory CD4^+^ T (TEM) cells (E), and germinal center (GC) B cells (F). Two experiments; *n* = 7 to 8. (G) Frequencies of gamma-interferon-positive (IFN-γ^+^) CD4^+^ T cells were measured in dLNs by intracellular cytokine staining following restimulation with *M. tuberculosis*. *n* = 8; two experiments. (H and I) CD8^+^ TCM (H) and CD8^+^ TEM (I) cells at 21 days postvaccination. (J) *Mycobacterium*-specific IgG in the serum at 21 days postvaccination. Three experiments; *n* = 9 to 11. Data from multiple experiments were analyzed using two-way ANOVA with Tukey’s multiple-comparison test. Unless indicated by brackets, *P* values compare with unvaccinated controls. *, *P* < 0.05; **, *P* < 0.01; ***, *P* < 0.001. (K) Heat map showing cluster analysis of differentially regulated (*P* < 0.05, 2-fold or greater difference) genes in dLNs of vaccinated mice (*n* = 4 to 5) versus naive controls (*n* = 4). Microarray data were analyzed using GeneSpring 12.6 GX (Agilent Technologies), with quality control filters, normalization, and one-way ANOVA. (R1) Cell cycle, mitosis, DNA replication/repair, proliferation. (R2) Antimicrobial responses, metabolism, proliferation. (R3) Regulation of apoptosis, endoplasmic reticulum (ER) genes, pathogen response, antibody production. (R4) IFN-induced genes. (R5) Inflammation, neutrophils, myeloid cells, adhesion. (R6) Lipid/glucose metabolism. (R7) Lipid binding, Wnt signaling network. (R8) Cell signaling (details in Table S1 in the supplemental material).

Because BCG Δ*ureC*::*hly* Δ*nuoG*-vaccinated mice had the lowest bacterial burdens following *M. tuberculosis* challenge, we aimed to determine which immune responses were associated with protection. The more rapid increase in size of dLNs of BCG Δ*ureC*::*hly* Δ*nuoG*-vaccinated mice in the days following vaccination suggested increased stimulation of the acquired immune response (Fig. 5A). Previously, we had demonstrated significantly increased Ag85B-specific central memory CD4^+^ T cells in dLNs of BCG Δ*ureC*::*hly*-vaccinated mice compared to BCG SSI-vaccinated mice at 14 days (6), and here, we observed the same trend, with similar numbers of Ag85B-specific CD4^+^ T cells in BCG Δ*ureC*::*hly* Δ*nuoG*-vaccinated mice at day 14 (Fig. 5B). In addition, at day 21, frequencies were significantly increased for T follicular helper cells (Fig. 5C), central memory and effector memory CD4^+^ T cells (Fig. 5D and E), germinal center B cells (Fig. 5F), and gamma interferon (IFN-γ)-producing CD4^+^ T cells (Fig. 5G) in dLNs of BCG Δ*ureC*::*hly* Δ*nuoG*-vaccinated mice compared to BCG SSI-vaccinated mice. No significant effect was found in frequencies of central memory and effector memory CD8^+^ T cells (Fig. 5H and I) in comparison to BCG SSI-vaccinated mice (Fig. 5B). Frequencies of T follicular helper cells, effector memory CD4^+^ T cells, and IFN-γ-producing CD4^+^ T cells were also increased in spleens of BCG Δ*ureC*::*hly* Δ*nuoG*-vaccinated mice compared to BCG SSI-vaccinated mice, whereas germinal center B cells, central memory CD4^+^ T cells, and central and effector memory CD8^+^ T cells were not significantly different (see Fig. S2A to G in the supplemental material). *Mycobacterium*-specific immunoglobulin G (IgG) levels (Fig. 5J) were markedly increased after vaccination with both recombinant strains compared to vaccination with the current vaccine strain BCG SSI. We have already observed higher antibody levels induced by BCG Δ*ureC*::*hly* than by BCG, both in mice and in humans (6, 8). Overall, the trend was qualitatively similar for the two recombinant BCG strains, but BCG Δ*ureC*::*hly* Δ*nuoG*-vaccinated mice had increased CD4^+^ T cell responses compared to BCG Δ*ureC*::*hly*-vaccinated mice, as well as increased germinal center B cells, suggesting synergism between the mechanisms of efficacy of the two genetic strain modifications.

### Gene expression analysis of dLNs in BCG Δ*ureC*::*hly*- and BCG Δ*ureC*::*hly* Δ*nuoG*-vaccinated mice.

Microarray analyses were performed to assess global host gene expression profiles in dLNs of mice in response to vaccination (Fig. 5K). Overall, results revealed earlier and stronger induction of immune responses by both recombinant BCG strains, particularly BCG Δ*ureC*::*hly* Δ*nuoG*, with vastly more genes differentially expressed (*P* < 0.05; fold change, >2) than after BCG vaccination (see Fig. S3A in the supplemental material). Genes in R1- to R8-labeled gene clusters are listed in Table S1 in the supplemental material. Because BCG Δ*ureC*::*hly* Δ*nuoG* was derived from BCG Δ*ureC*::*hly*, numerous genes showed similar expression patterns in response to the two vaccine strains compared to BCG, e.g., *IL-1β* and *IL-18*, previously found to be upregulated 1 day after BCG Δ*ureC*::*hly* vaccination (7) (see Fig. S3B and C). In contrast, gene expression levels of IFN-inducible GTPases (Gbps, Irgs, and Givns), often associated with phagosomal or autophagic vacuoles and inflammasome activation (25–28), and ubiquilin, a key player in xenophagic responses to *M. tuberculosis* (13), were increased at earlier time points and to greater levels in BCG Δ*ureC*::*hly* Δ*nuoG*-vaccinated mice (see Fig. S3C), which is in line with the observed increase in bacterium-associated LC3 responses *in vitro.* Table S2 in the supplemental material lists genes significantly upregulated (*P* < 0.05) ≥2-fold versus the naive group specifically in BCG Δ*ureC*::*hly* Δ*nuoG*-immunized mice at days 1, 3, and 7 postvaccination. By day 7, expression of IFN-inducible GTPases had also increased in BCG- and BCG Δ*ureC*::*hly*-vaccinated mice but tended to remain slightly lower than that after BCG Δ*ureC*::*hly* Δ*nuoG* vaccination in most cases (see Fig. S4). Early upregulation of genes for inflammasome-associated interleukin-1β (IL-1β), IL-18, cytosolic DNA sensor Ifi204, and Gbps was confirmed by reverse transcription-PCR (RT-PCR) (see Fig. S5). Gene ontology (GO) analysis of differentially expressed genes highlighted involvement of acute inflammatory responses at day 1 postvaccination and immune cell activation and differentiation at day 3, while concurrently with enhanced dLN enlargement in BCG Δ*ureC*::*hly* Δ*nuoG*-vaccinated mice, cell cycle and developmental pathways featured prominently at days 3 and 7 (see Table S3). Due to the overwhelming dominance of cell cycle and tissue development pathways obtained in GO analysis of day 7 gene expression, only the top 20 pathways are listed in the table.

## DISCUSSION

An estimated 9.6 million new active TB cases and 1.5 million deaths occurred in 2014 (1), emphasizing the need for a more efficacious vaccine. The current TB vaccine, BCG, shows variable efficacy against the pulmonary form of the disease, although it has 60 to 80% protective efficacy against severe disseminated forms of disease in infants, such as meningitis (29). A recombinant live vaccine, BCG Δ*ureC*::*hly*, which expresses listeriolysin, is the most advanced BCG replacement vaccine candidate in clinical trials, having completed phase I and phase IIa safety and immunogenicity trials successfully (NCT01479972, NCT01113281, and NCT00749034) and currently undergoing a phase II safety and immunogenicity trial in HIV-exposed newborns (NCT02391415). Our previous studies suggest that increased preclinical efficacy of BCG Δ*ureC*::*hly* is based on (i) high egression of BCG-derived protein antigens and (ii) release of bacterial DNA into the host cell cytosol, subsequent induction of apoptosis and inflammasome activation, and increased generation of central memory CD4^+^ T cell responses (6–8). While this vaccine awaits phase IIb efficacy trials, next-generation vaccines are being designed and tested in preclinical models aimed at optimizing efficacy and/or safety.

Recently, an antiapoptotic virulence gene, *nuoG*, was identified in *M. tuberculosis* (18, 19). As apoptosis is thought to enhance adaptive immune responses through cross-presentation (17, 20, 21), we aimed to augment the efficacy and safety of BCG Δ*ureC*::*hly* by deleting *nuoG.* Because BCG Δ*ureC*::*hly* already induces 100-fold-better protection than the sham control and 10-fold protection over BCG in mice, which has not been achieved by other recombinant BCG vaccine candidates to date (5, 24), it sets a high bar for further improvement. Yet, our results demonstrate that vaccination with BCG Δ*ureC*::*hly* Δ*nuoG* further increased protection about 5-fold in lungs of mice challenged with the *M. tuberculosis* laboratory strain (H37Rv) and a clinical *M. tuberculosis* isolate (Beijing/W lineage) at 90 and 180 days p.i. while maintaining excellent safety in immunodeficient SCID mice. Note that increased protection was sustained against the clinical isolate *M. tuberculosis* Beijing/W, which is considered notoriously resistant against BCG vaccination. Deletion of *nuoG* from unmodified BCG also resulted in decreased pulmonary pathogen loads, suggesting a standalone function for *nuoG* in protective efficacy.

The increased efficacy of BCG Δ*ureC*::*hly* Δ*nuoG* versus BCG Δ*ureC*::*hly* was associated with a numerical increase in CD4^+^ T_EM_ cells, T_FH_ cells, and germinal center B cells and a trend toward an increase of CD4^+^ T_CM_ cells. T_EM_ cells, which appear early after infection and can secrete effector cytokines such as IFN-γ and tumor necrosis factor alpha (TNF-α), provide immediate protection, while T_CM_ cells proliferate in the LN and generate new waves of effector cells upon reexposure to antigen (6, 30, 31). Recently, T_CM_ cells were found to be associated with protection after vaccination (6, 31). We demonstrated previously that vaccination with BCG Δ*ureC*::*hly* increased T_CM_ responses as well as T_FH_ responses and antibody production (6), and these responses seem to be further enhanced after BCG Δ*ureC*::*hly* Δ*nuoG* vaccination. Transfer studies demonstrated that protection against TB was conferred by the T_CM_ cell population (6). Both T_CM_ and T_FH_ express CXCR5, and CXCR5-expressing T cells have previously been correlated with decreased lung pathology following vaccination and challenge with *M. tuberculosis* (32). The T_FH_ population, which decreases more quickly than the long-lived T_CM_ population, stimulates germinal center B cell responses (33), but the role of B cells and antibodies in TB remains unclear. Both T_CM_ and T_FH_ cells have been associated with enhanced antibody responses (30). Vaccination with BCG Δ*ureC*::*hly* and BCG Δ*ureC*::*hly* Δ*nuoG* leads to increased antibody responses in mice, and enhanced production of *Mycobacterium*-specific antibodies was found in a phase I clinical trial in the BCG Δ*ureC*::*hly* group over the BCG group (8). Although it is difficult to foresee a protective role of antibodies to *M. tuberculosis* once it is hidden inside host cells, vaccine-induced preexisting antibodies could participate in prevention of infection with *M. tuberculosis* (34). Apart from their role in antibody production, B cells can also present antigen to T cells and enhance T_CM_ and T_FH_ cell development (35).

In accordance with improved immune responses, dLNs were found to increase in size earlier in BCG Δ*ureC*::*hly* Δ*nuoG*-vaccinated mice*.* Transcriptome analysis revealed similar changes in gene expression in both BCG Δ*ureC*::*hly*- and BCG Δ*ureC*::*hly* Δ*nuoG*-vaccinated mice, with induction of genes such as *IL-1β*, *IL-18*, Gbps, and other GTPases, although the expression of genes associated with GTPase activity, intracellular resistance, inflammatory responses, cell activation, and cell proliferation tended to be higher in BCG Δ*ureC*::*hly* Δ*nuoG*-vaccinated mice. Genes significantly differentially expressed between BCG Δ*ureC*::*hly* Δ*nuoG*- and BCG Δ*ureC*::*hly*-vaccinated mice included *Ifng*- and IFN-γ-induced genes, suggesting an improved antimicrobial Th1-type response. Overall, *nuoG* deletion appeared to synergize with, and enhance, the protective effects of the Δ*ureC*::*hly* mutation in BCG against *M. tuberculosis*, since most responses were quantitatively but not qualitatively different*.* Unexpectedly, the present study also uncovered a novel potential role for the mycobacterial gene *nuoG* in suppressing host cell LC3-mediated pathways, in addition to its previously reported role in inhibition of apoptosis (18, 19). For analysis of *nuoG*-mediated effects, we employed THP-1 cells, shown to be an appropriate model for human alveolar macrophage responses to mycobacterial infection (36) and used for investigation of antiapoptotic functions of *nuoG* (18, 19). Previously, we have shown that BCG Δ*ureC*::*hly* induced increased overall levels of the autophagy marker LC3 in infected THP-1 cells in an AIM2- and STING-dependent manner compared to BCG SSI (7). However, colocalization of LC3 with bacteria was not observed. Here, we demonstrate that both BCG and BCG Δ*ureC*::*hly* strains deficient in *nuoG* colocalized with LC3 within THP-1 cells, which did not occur in the parental strains. This effect was observed to begin between 4 and 8 h p.i.; at 24 h p.i., most bacteria were completely surrounded by LC3, and up to 48 h p.i., LC3 was still associated with the bacteria. It has been shown that artificially inducing autophagy during BCG vaccination increases antigen processing, leading to improved Th1 responses and vaccine efficacy (14). It remains to be seen whether the strong colocalization of the autophagy protein LC3 mediates enhanced destruction of the bacteria through canonical autophagic pathways or through LAP pathways (22) and whether this provides a link to the enhanced protective immune responses seen in BCG Δ*ureC*::*hly* Δ*nuoG*-vaccinated mice. During autophagy, proteins such as LC3 form a double-membraned autophagosome, which captures cytoplasmic components and transports them to the lysosome for degradation (37). During LAP, autophagy components such as LC3 are translocated to the phagosomal membrane and promote fusion with the lysosome, which does not involve the formation of a double-membrane autophagosome (22). Conjugation of LC3 to phagosomes and subsequent association with lysosomes require the activity of NADPH oxidase (NOX2) and the production of reactive oxygen species (ROS). Intriguingly, previous studies on *nuoG* in *M. tuberculosis* revealed that inhibition of apoptosis was related to its ability to neutralize NOX2-dependent ROS (18). Therefore, the ability of *nuoG* to neutralize NOX2-dependent ROS could also impact the induction of LAP, explaining why *nuoG*-deficient BCG has increased LC3 colocalized to phagocytosed bacteria. NOX2 activity is also required for efficient cross-presentation by human dendritic cells (38). Therefore, we speculate that the *nuoG* gene of *M. tuberculosis* plays multiple roles in inhibiting optimal host immune responses and antigen presentation.

The mechanism by which *nuoG* deletion leads to increased targeting of the bacteria is curious, as it implies a role for *nuoG* in inhibiting LAP, and possibly other autophagic pathways involving LC3. However, BCG Δ*ureC*::*hly* and BCG Δ*ureC*::*hly* Δ*nuoG* had similarly decreased survival times and less dissemination in immunocompetent mice, suggesting that inflammasome- or apoptosis-mediated mechanisms induced by both vaccine strains are primarily responsible for eliminating BCG. Apoptosis of infected macrophages is considered important for immunity to pulmonary TB (18–20, 39). Similarly to *M. tuberculosis* (19), we found that BCG did not induce elevated levels of apoptosis in THP-1 macrophages, and deletion of *nuoG* did not enhance this. As *nuoG*-inhibited apoptosis in *M. tuberculosis* relies on neutralization of NOX2-dependent ROS (18), it is possible that *nuoG*-deficient BCG induces lower levels of ROS. *In vivo*, apoptosis was increased only at day 14 in mice vaccinated with BCG Δ*ureC*::*hly* Δ*nuoG*, suggesting that increased apoptosis occurs downstream of altered intracellular mechanisms initiated in the absence of *nuoG*, including increased oxidative stress or autophagy.

In summary, our data reveal a potential novel role for mycobacterial *nuoG* in inhibition of LC3-mediated autophagic pathways, with relevance for protective immunity against *M. tuberculosis*. Even though the prominent long-term protective efficacy of BCG Δ*ureC*::*hly* of up to 2 logs over that of BCG set the bar high for further improvement, we demonstrate a significant increase in vaccine efficacy as reflected by a 5-fold-lower pulmonary *M. tuberculosis* burden upon deletion of *nuoG* from BCG Δ*ureC*::*hly*, corresponding with enhanced immune responses after vaccination and paralleled by an excellent safety profile.

## MATERIALS AND METHODS

### Bacterial strains and growth conditions.

*M. tuberculosis* H37Rv (American Type Culture Collection; catalog no. 27294), *M. tuberculosis* Beijing/W (RIVM catalog no. 17919; isolated in Mongolia), BCG Danish 1331 (BCG SSI) (American Type Culture Collection; catalog no. 35733), BCG Δ*ureC*::*hly* (5), and derivatives were grown in Middlebrook 7H9 broth (Becton, Dickinson) supplemented with albumin-dextrose-catalase enrichment (Becton, Dickinson), 0.2% glycerol, and 0.05% Tween 80 or on Middlebrook 7H11 agar (Becton, Dickinson) containing 10% (vol/vol) oleic acid-albumin-dextrose-catalase enrichment (Becton, Dickinson) and 0.2% glycerol. Cultures were grown to mid-log phase in 1-liter roller bottles at 37°C and 2 rpm. For vaccine stock preparations, log-phase bacilli were harvested, washed with phosphate-buffered saline (PBS), and stored at −80°C in PBS-10% glycerol. Prior to vaccination, vials were thawed, and cells were pelleted and administered as PBS suspensions. For CFU estimation, serial dilutions were performed in PBS-0.05% Tween 80 (PBST) and dilutions were plated on Middlebrook 7H11 agar. Plates were incubated at 37°C for 3 to 4 weeks prior to CFU counting.

### Generation of recombinant BCG strains.

The *nuoG* gene of BCG or BCG Δ*ureC*::*hly* was disrupted by site-directed mutagenesis. One-kilobase fragments flanking *nuoG* were amplified by using PCR and specific oligonucleotides ko5′nuoG.fwd (5′ ATCTTAAGTACGCGGTGAGGTGGTG 3′)/ko5′nuoG.rev (5′ TATCTAGAATTTCGACGCCGTCGAT 3′) and ko3′nuoG.fwd (5′ ATAAGCTTTCAATCACCTTGCCGTG 3′)/ko3′nuoG.rev (5′ TAACTAGTCTCGGCGAGCATGAACA 3′) (restriction sites underlined) and inserted into pYUB854 (40). The knockout plasmid was then electroporated into BCG or BCG Δ*ureC*::*hly*, and transformants were selected on Middlebrook 7H11 agar supplemented with 80 µg/ml hygromycin B. The resistance cassette was subsequently removed by standard methods described previously (41). Site-directed mutagenesis and resistance marker removal were confirmed by automated sequencing of the *nuo* operon.

### *In vitro* apoptosis and autophagy assays.

Cells of the human monocytic cell line THP-1 (American Type Culture Collection; catalog no. TIB202; authenticated; tested for *Mycoplasma* contamination) were cultured in RPMI 1640 medium (Gibco) containing 10% heat-inactivated fetal bovine serum, 10 mM HEPES buffer solution, 1 mM sodium pyruvate, 2 mM l-glutamine, and penicillin-streptomycin (Pen-Strep). THP-1 monocytic cells were differentiated into macrophages in RPMI medium containing 40 ng/ml phorbol myristate acetate (PMA) (Sigma) for 48 h. BCG, BCG Δ*nuoG*, BCG Δ*ureC*::*hly* BCG, and BCG Δ*ureC*::*hly* Δ*nuoG* were labeled red with the PKH26 linker kit (Sigma) and used to infect THP-1 macrophages (multiplicity of infection [MOI] of 10, 20, and 100) for 4 h. After 4 h, cells were washed 3 times with PBS to eliminate extracellular BCG and fresh medium was added. For apoptosis, the assays were performed in 96-well plates. After 24 and 48 h, activated caspases were labeled with Cell Event Caspase-3/7 Green detection reagent with the NucRed Live 647 ReadyProbes Reagent-1 kit (Life Technologies) for nuclear staining. For autophagy analysis, the THP-1 macrophages were differentiated and infected on coverslips. After 4, 8, 24, and 48 h, treated cells were washed with PBS and fixed with 4% paraformaldehyde (Electron Microscopy Sciences). Fixed cells were washed with PBS, permeabilized with 0.3% Triton X-100, and blocked with 1% bovine serum albumin (BSA). Cells were incubated with anti-LC3-II antibody (Sigma L8918) at room temperature for 1 h and then incubated with Alexa 488 anti-rabbit IgG antibody (Life Technologies) for 30 min. Coverslips were mounted using Hoechst mounting medium and evaluated on a confocal microscope under oil with a 63× lens (Leica TCS SP-8). For quantification, 300 individual infected cells were evaluated for each BCG strain in groups of 25, and the percentage of the 25 cells showing LC3 colocalization with bacteria was plotted as one point.

### Immune responses.

Lymph nodes, spleens, and blood of vaccinated C57BL/6 mice were collected at day 21. Single-cell suspensions were generated from lymph nodes and spleens in Iscove's modified Dulbecco's medium (IMDM) 10% fetal calf serum (FCS) Pen-Strep, and flow cytometry was performed to quantify cell populations. Details of antibodies used are as follows: T cell panel, CD3 Alexa 700 (eBioscience; clone 17A2), CD4 phycoerythrin (PE)-Cy7 (BioLegend; clone RM4-5), CD8 V500 (BD Horizon, clone 53-6.7), CD62L allophycocyanin (APC) (BD Pharmingen; clone MEL-14), CD44 Pacific Blue (in-house, clone IM7), CXCR5 PE (BD Pharmingen; clone 2G8), CCR7 peridinin chlorophyll protein (PerCP) (BioLegend; clone 4B12), PD-1 fluorescein isothiocyanate (FITC) (BioLegend; clone 29F-1A12); B cell panel, B220 V500 (BD Horizon; clone RA3-6B2), CD138 APC (BioLegend; clone 281-2), Fas-biotin (eBioscience; clone 15A7) with PerCP streptavidin (BD Bioscience), GL7 FITC (BD Bioscience; clone GL7), major histocompatibility complex II (MHC-II) Pacific Blue (BioLegend; clone M5/114.15.2). Central memory CD4^+^ T cells were CD3^+^ CD4^+^ CD44^high^ CD62^high^, effector memory CD4^+^ T cells were CD3^+^ CD4^+^ CD44^high^ CD62L^low^, T follicular helper cells were CD44^high^ CD62^low^ CCR7^low^ PD-1^+^, and germinal center B cells were B220^+^ GL7^+^ Fas^+^. For tetramer-based analysis of antigen-specific T cells, I-Ab:Ag85B (positions 280 to 294; FQDAYNAAGGHNAVF) tetramers were obtained from the National Institutes of Health (NIH) tetramer facility (Bethesda, MD). A total of 10 nM Ag85B tetramer was added to single-cell suspensions of pooled spleen and lymph nodes (LNs; cervical, retromaxillary, inguinal, peripheral, and mesenteric) for 1 h at room temperature. Tetramer^+^ cells were enriched as described elsewhere (42). In brief, samples were incubated with magnetic antifluorochrome microbeads and concentrated by an LS column (Miltenyi Biotec), and the resulting cell fractions were analyzed by flow cytometry. For intracellular cytokine production, lymph node cells and splenocytes were plated overnight with or without 10 µg/ml *M. tuberculosis* H37Rv lysate (BEI Resources; NR-14822) at 37°C. Brefeldin (Sigma) was added to lysate-incubated samples, and PMA-ionomycin-brefeldin (all from Sigma) were added to cells incubated with medium alone, for 4 h at 37°C. Following this, cells were stained for surface markers, fixed with 2% paraformaldehyde, permeabilized with saponin buffer (saponin, 1 g/liter; CaCl_2_, 0.11 g/liter; MgSO_4_, 0.125 g/liter; NaN_3_, 0.5 g/liter; bovine serum albumin [BSA] 1 g/liter; 10 mM HEPES in PBS, pH 7.4), and stained for intracellular cytokine production. The intracellular cytokine panel consisted of CD3 Alexa 700 (eBioscience; catalog no. 56-0032082), CD4 Pacific Blue (BD Pharmingen; catalog no. 588107), CD8 PerCP, and IFN-γPE-Cy7. Samples were acquired on a fluorescence-activated cell sorting (FACS) LSR II cytometer (BD Biosciences) using BD FACS Diva software and analyzed using FlowJo v10 (TreeStar). *Mycobacterium*-specific antibodies in serum were measured by indirect enzyme-linked immunosorbent assay (ELISA) using *M. tuberculosis* H37Rv lysate (BEI Resources; NR-14822) and anti-mouse IgG alkaline phosphatase (AP) (SouthernBiotech).

### Histology and *ex vivo* apoptosis assays.

Lymph nodes were collected at days 0, 3, 7, and 14 postvaccination. Tissues were fixed in 4% formaldehyde in PBS and embedded in paraffin wax. Tissue sections were stained with hematoxylin and eosin (H&E), and terminal deoxynucleotidyltransferase-mediated dUTP-biotin nick end labeling (TUNEL) staining was performed for apoptosis (APO-BrdU TUNEL staining kit; Life Technologies). Apoptotic cells were counted per field of view at ×200 magnification.

### Transcriptome analysis.

Mice were vaccinated with BCG SSI, BCG Δ*ureC::hly*, or BCG Δ*ureC::hly* Δ*nuoG*, and lymph nodes were collected in RNAlater RNA stabilization reagent (Qiagen) at days 1, 3, and 7 postvaccination. Following the collection of all lymph nodes, samples were removed from RNAlater, homogenized in Trizol (Qiagen) using a gentleMACS dissociator (Miltenyi), and then frozen at −80°C. After thawing, samples were allowed to stand at room temperature for 5 min, and then precipitation was performed using isopropanol, ammonium acetate (Ambion AM9070G), and glycogen (Ambion AM9510), and pellets were washed with 70% ethanol and resuspended in RNase-free water on ice. The concentrations were measured on a NanoDrop spectrophotometer, and the quality of the RNA was assessed using a Bioanalyzer. Agilent whole-mouse-genome microarrays were performed using RNA samples labeled with a one-color Quick Amp labeling kit (Agilent Technologies) according to the manufacturer’s instructions. To avoid batch-specific effects, we spread samples from different groups and time points between microarray chips. Scanning of microarrays was performed with 3-µm resolution and 20-bit image depth using a G2565CA high-resolution laser microarray scanner (Agilent Technologies). Microarray image data were analyzed and extracted with the Image Analysis/Feature Extraction software G2567AA v.A.11.5.1.1 (Agilent Technologies) using the protocol GE1_1105_Oct12 and recommended settings. Analysis of transcripts obtained at day 1 after vaccination with BCG and BCG Δ*ureC*::*hly* only, in comparison to naive mice, was previously performed and published (7). Here, microarray data obtained from all days and with all vaccine strains were analyzed using GeneSpring 12.6 GX (Agilent Technologies), with quality control filters, normalization, and one-way analysis of variance (ANOVA). Naive mice were selected as the control group for comparative analysis. The *P* values were corrected for multiple comparisons, and values of *P* < 0.05 were considered statistically significant. Subgroups of differentially expressed genes with change greater than 2-fold from the comparison group (*P* < 0.05) were used for generation of heat maps, Venn diagrams, pathway analysis, and GO analysis. RT-PCR was performed to validate expression of selected genes. cDNA was generated by reverse transcription using the iScript cDNA synthesis kit (770-8897; Bio-Rad), according to the manufacturer’s instructions, on a Gene Amp PCR System 9700 machine (Applied Biosystems). PCR was performed on a Step One Plus real-time PCR machine (Applied Biosystems) using the SYBR green Fast mix (Thermo Fisher Scientific) with the Fast protocol and the primers listed in Table S4 in the supplemental material. Primers were designed using Primer3Plus software (43). Analysis was performed using the threshold cycle (*C_T_*) comparative method (44), with the housekeeping gene *Ywhaz* used for normalization.

### Animal experimentation.

Nine- to 10-week-old female mice (BALB/c and CB-17/Icr-*Prkdc*^SCID^/Rj [Janvier]; C57BL/6 [Charles River Laboratories]) were kept under specific-pathogen-free conditions in groups of five in individually ventilated cages. Animals were vaccinated subcutaneously in the tail base with 10^6^ CFU of BCG strains. At designated time points postvaccination, mice were euthanized and tissues of interest were removed and homogenized in PBS-0.05% Tween 80 prior to CFU enumeration or processed otherwise. For protective efficacy studies, mice were aerosol challenged 90 days postvaccination with a low dose of 100 to 200 CFU of *M. tuberculosis*. All animal studies have been ethically reviewed and approved by the State Office for Health and Social Services, Berlin, Germany. Experimental procedures were carried out in accordance with the European directive 2010/63/EU on Care, Welfare and Treatment of Animals.

### Statistical methods.

GraphPad Prism 6.04 (GraphPad Software, Inc.) was used for statistical analysis. Survival curves were calculated by using the Mantel-Cox log rank test. Vaccine efficacy was evaluated by one-way ANOVA with Tukey’s multiple-comparison test. Similarity of variances between groups compared was determined by the Brown-Forsythe test. For autophagy quantification, the Mann-Whitney test was used for pairwise comparison. Two-way ANOVA with Tukey’s multiple-comparison test was used to evaluate immunology data.

### Microarray data accession number.

Microarray data are available from the NCBI GEO database under accession code GSE74282.

## SUPPLEMENTAL MATERIAL

Figure S1  Dissemination and survival of BCG and recombinant derivatives in mice. Animals were subcutaneously vaccinated with 10^6^ CFU of BCG SSI, BCG Δ*ureC*::*hly*, or BCG Δ*ureC*::*hly* Δ*nuoG* (Fig. 3A). The dissemination of live vaccines was determined by plating organ homogenates on agar at designated time points. When bacterial counts were expected to be low, the entire organ homogenate was plated. Error bars for data points at log_10_ = 1 and below are not included as they were negative. Data points represent means and standard deviations (*n* = 5). One representative experiment out of 3 is shown. LN, lymph node. Download Figure S1, PDF file, 0.1 MB

Figure S2  Immune responses in spleens after vaccination with BCG, BCG Δ*ureC*::*hly*, and BCG Δ*ureC*::*hly* Δ*nuoG*. (A to F) Frequencies of T follicular helper (TFH) cells (A), germinal center (GC) B cells (B), CD4^+^ central memory T (TCM) cells (C), effector memory T (TEM) cells (D), CD8^+^ TCM cells (E), and TEM cells (F) at 21 days postvaccination. Two experiments; *n* = 7 to 8. (G) Frequencies of IFN-γ^+^ CD4^+^ T cells were measured in lymph nodes by intracellular cytokine staining following restimulation with *M. tuberculosis* H37Rv lysate. Two experiments; *n* = 8. Data were analyzed using two-way ANOVA with Tukey’s multiple-comparison test. *, *P* < 0.05; **, *P* < 0.01; ***, *P* < 0.001. Download Figure S2, PDF file, 0.1 MB

Figure S3  Differential gene expression after vaccination with BCG, BCG Δ*ureC*::*hly*, and BCG Δ*ureC*::*hly* Δ*nuoG*. (A) Microarray analysis demonstrates that vaccination of mice with BCG Δ*ureC*::*hly* and particularly BCG Δ*ureC*::*hly* Δ*nuoG* drives earlier and stronger immune responses in the lymph nodes compared to BCG SSI. Number of entities significantly differentially regulated at least 2-fold (*P* < 0.05) in whole-genome Agilent mouse arrays after vaccination with different BCG strains, compared to the naive controls (*n* = 4 to 5 per group). Microarray data were analyzed using GeneSpring 12.6 GX (Agilent Technologies), with quality control filters, normalization, and one-way ANOVA. (B) Venn diagrams determining overlap between significantly upregulated entities (>2-fold change, *P* < 0.05) after vaccination with different BCG strains. (C) Entities significantly upregulated 3 days after both BCG Δ*ureC*::*hly* and BCG Δ*ureC*::*hly* Δ*nuoG* vaccination include molecules associated with inflammasome activation, while BCG Δ*ureC*::*hly* Δ*nuoG* vaccination led to significantly stronger expression of IFN-inducible GTPases and xenophagy-associated genes. *, *P* < 0.05; **, *P* < 0.01; ***, *P* < 0.001; ****, *P* < 0.0001. Download Figure S3, PDF file, 0.4 MB

Figure S4  Differential expression of IFN-inducible GTPases 7 days after vaccination with BCG, BCG Δ*ureC*::*hly*, and BCG Δ*ureC*::*hly* Δ*nuoG*. Draining lymph nodes were collected at day 7 postvaccination, and gene expression was analyzed by whole-genome mouse microarray. Microarray data were analyzed using GeneSpring 12.6 GX (Agilent Technologies), with quality control filters, normalization, and one-way ANOVA. *, *P* < 0.05; **, *P* < 0.01; ***, *P* < 0.001; ****, *P* < 0.0001. PBS, phosphate-buffered saline. Download Figure S4, PDF file, 0.1 MB

Figure S5  Upregulation of inflammasome-associated genes *IL-1β*, *IL-18*, and *Ifi204* and Gbps at day 3 was validated by RT-PCR. RNA from lymph nodes of mice vaccinated with BCG, BCG Δ*ureC*::*hly*, and BCG Δ*ureC*::*hly* Δ*nuoG* was reverse transcribed to cDNA, and PCRs were performed on a Step One Plus real-time PCR machine. Analysis was performed using the comparative *C_T_* method with *Ywhaz* used as a housekeeping gene, and values from lymph nodes of vaccinated groups (*n* = 5) were compared to the average value of the naive control group (*n* = 5). Results are shown as fold difference compared to the average value of the naive control group. Significant differences are indicated by asterisks (*, *P* < 0.05; **, *P* < 0.01; ***, *P* < 0.001) in comparison to the BCG-vaccinated group, unless otherwise indicated by brackets. Download Figure S5, PDF file, 0.1 MB

Table S1  Lists of genes in the heat map gene clusters labeled R1 to R8. A heat map was generated in GeneSpring from genes significantly differentially regulated (*P* < 0.05) compared to naive controls, and lists were compiled of gene clusters. Long noncoding RNAs are not included. Confirmed or potential gene/protein functions were obtained from the Mouse Gene Detail (MGI) (http://www.informatics.jax.org), GeneCards (http://www.genecards.org/), National Center for Biotechnology Information (NCBI) (http://www.ncbi.nlm.nih.gov/), and UniProt (http://www.uniprot.org) online repositories. Table S1, DOCX file, 0.1 MB

Table S2  Genes significantly upregulated (*P* < 0.05) at least 2-fold compared to naive control only in BCG Δ*ureC*::*hly* Δ*nuoG*-vaccinated mice. Venn diagrams were plotted in GeneSpring to analyze overlap of genes significantly upregulated (*P* < 0.05) at least 2-fold compared to naive control in groups vaccinated with different strains of BCG, at 1, 3, and 7 days postvaccination. Lists of genes upregulated specifically in BCG Δ*ureC*::*hly* Δ*nuoG* mice are given here with their potential or confirmed protein functions. Noncoding genes and confirmed pseudogenes were not included. Gene/protein functions were obtained from the Mouse Gene Detail (MGI) (http://www.informatics.jax.org), GeneCards (http://www.genecards.org/), National Center for Biotechnology Information (NCBI) (http://www.ncbi.nlm.nih.gov/), and UniProt (http://www.uniprot.org) online repositories. The *P* values corrected for multiple comparisons are shown. Table S2, DOC file, 0.3 MB

Table S3  GO enrichment analysis of genes identified as significantly upregulated (*P* < 0.05) at least 2-fold compared to naive controls only in BCG Δ*ureC*::*hly* Δ*nuoG*-vaccinated mice. GeneSpring was used to perform GO analysis on genes significantly upregulated compared to naive controls in BCG Δ*ureC*::*hly* Δ*nuoG* mice only, as identified by Venn diagrams. At day 1, GO terms indicated involvement of genes involved in acute inflammation, while at day 3 they indicated immune activation and host defense processes as well as cell proliferation and differentiation. By day 7, developmental and cell cycle processes dominated the list of GO terms in the enlarged lymph node, and so only the first 20 hits are included in the list. The *P* values corrected for multiple comparisons are shown. Table S3, DOC file, 0.1 MB

Table S4 Primers for RT-PCR. Table S4, DOCX file, 0.1 MB
