# Supplementary figures and images for: Deletion of nuoG from the Vaccine Candidate Mycobacterium bovis BCG ΔureC::hly Improves Protection against Tuberculosis
Source: mBio. 2016 May 24;7(3):e00679-16. doi: 10.1128/mBio.00679-16 (PMC4895111; doi:10.1128/mBio.00679-16)

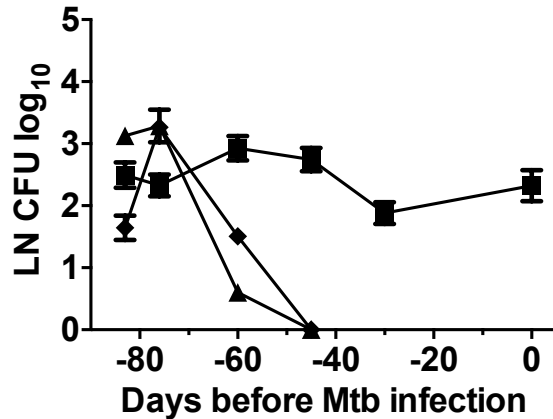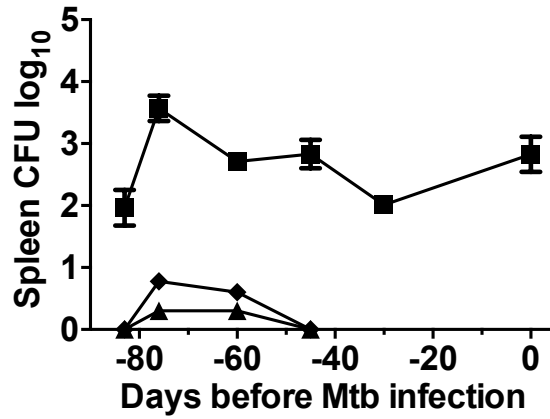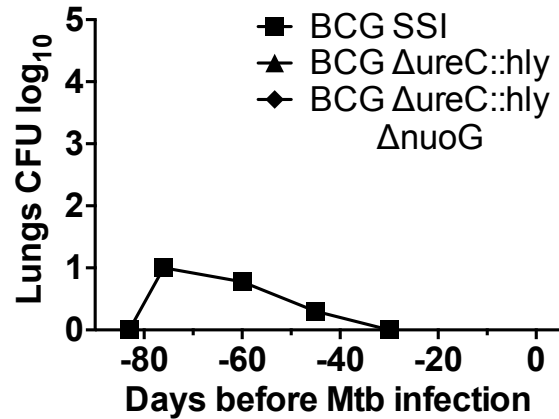

Supplement: Figure S1 — Dissemination and survival of BCG and recombinant derivatives in mice. Animals were subcutaneously vaccinated with 106 CFU of BCG SSI, BCG ΔureC::hly, or BCG ΔureC::hly ΔnuoG (Fig. 3A). The dissemination of live vaccines was determined by plating organ homogenates on agar at designated time points. When bacterial counts were expected to be low, the entire organ homogenate was plated. Error bars for data points at log10 = 1 and below are not included as they were negative. Data points represent means and standard deviations (n = 5). One representative experiment out of 3 is shown. LN, lymph node. Download [file mbo003162827sf1.pdf]

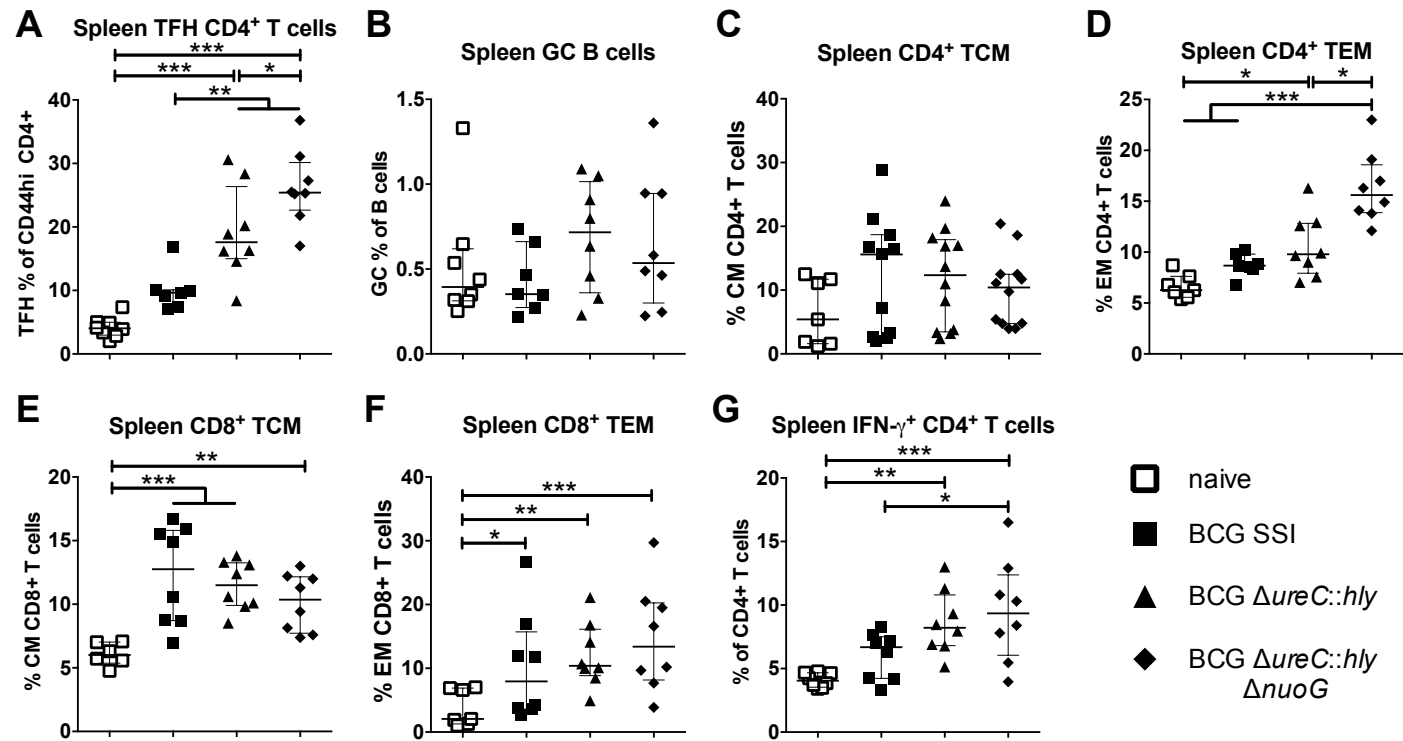

Supplement: Figure S2 — Immune responses in spleens after vaccination with BCG, BCG ΔureC::hly, and BCG ΔureC::hly ΔnuoG. (A to F) Frequencies of T follicular helper (TFH) cells (A), germinal center (GC) B cells (B), CD4+ central memory T (TCM) cells (C), effector memory T (TEM) cells (D), CD8+ TCM cells (E), and TEM cells (F) at 21 days postvaccination. Two experiments; n = 7 to 8. (G) Frequencies of IFN-γ+ CD4+ T cells were measured in lymph nodes by intracellular cytokine staining following restimulation with M. tuberculosis H37Rv lysate. Two experiments; n = 8. Data were analyzed using two-way ANOVA with Tukey’s multiple-comparison test. *, P < 0.05; **, P < 0.01; ***, P < 0.001. Download [file mbo003162827sf2.pdf]

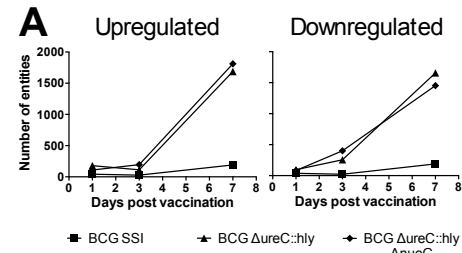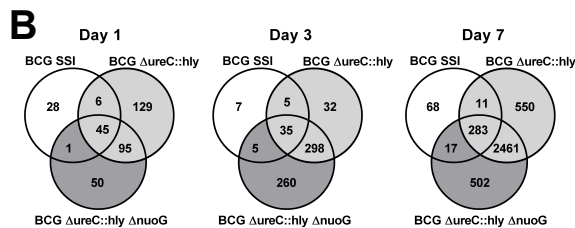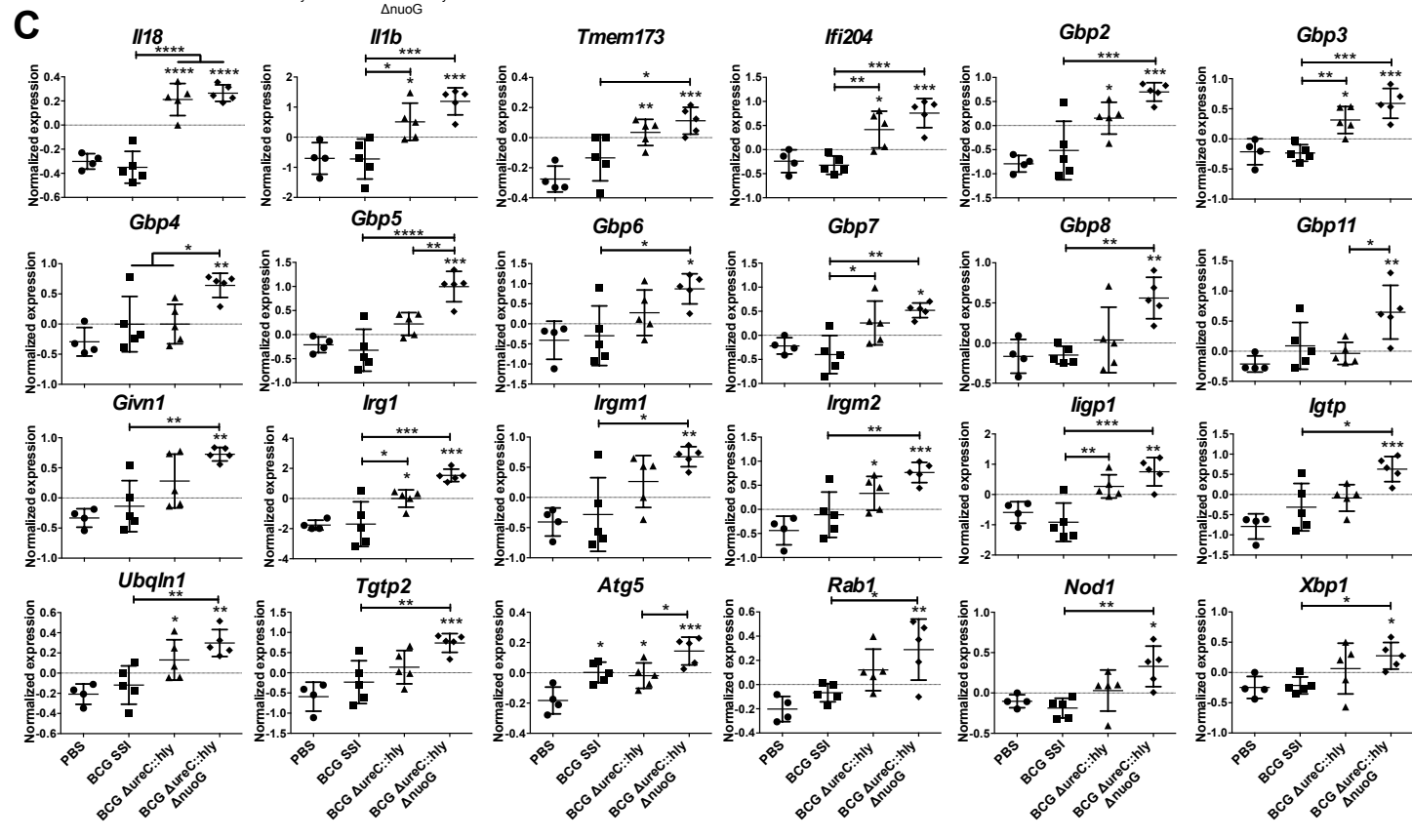

Supplement: Figure S3 — Differential gene expression after vaccination with BCG, BCG ΔureC::hly, and BCG ΔureC::hly ΔnuoG. (A) Microarray analysis demonstrates that vaccination of mice with BCG ΔureC::hly and particularly BCG ΔureC::hly ΔnuoG drives earlier and stronger immune responses in the lymph nodes compared to BCG SSI. Number of entities significantly differentially regulated at least 2-fold (P < 0.05) in whole-genome Agilent mouse arrays after vaccination with different BCG strains, compared to the naive controls (n = 4 to 5 per group). Microarray data were analyzed using GeneSpring 12.6 GX (Agilent Technologies), with quality control filters, normalization, and one-way ANOVA. (B) Venn diagrams determining overlap between significantly upregulated entities (>2-fold change, P < 0.05) after vaccination with different BCG strains. (C) Entities significantly upregulated 3 days after both BCG ΔureC::hly and BCG ΔureC::hly ΔnuoG vaccination include molecules associated with inflammasome activation, while BCG ΔureC::hly ΔnuoG vaccination led to significantly stronger expression of IFN-inducible GTPases and xenophagy-associated genes. *, P < 0.05; **, P < 0.01; ***, P < 0.001; ****, P < 0.0001. Download [file mbo003162827sf3.pdf]

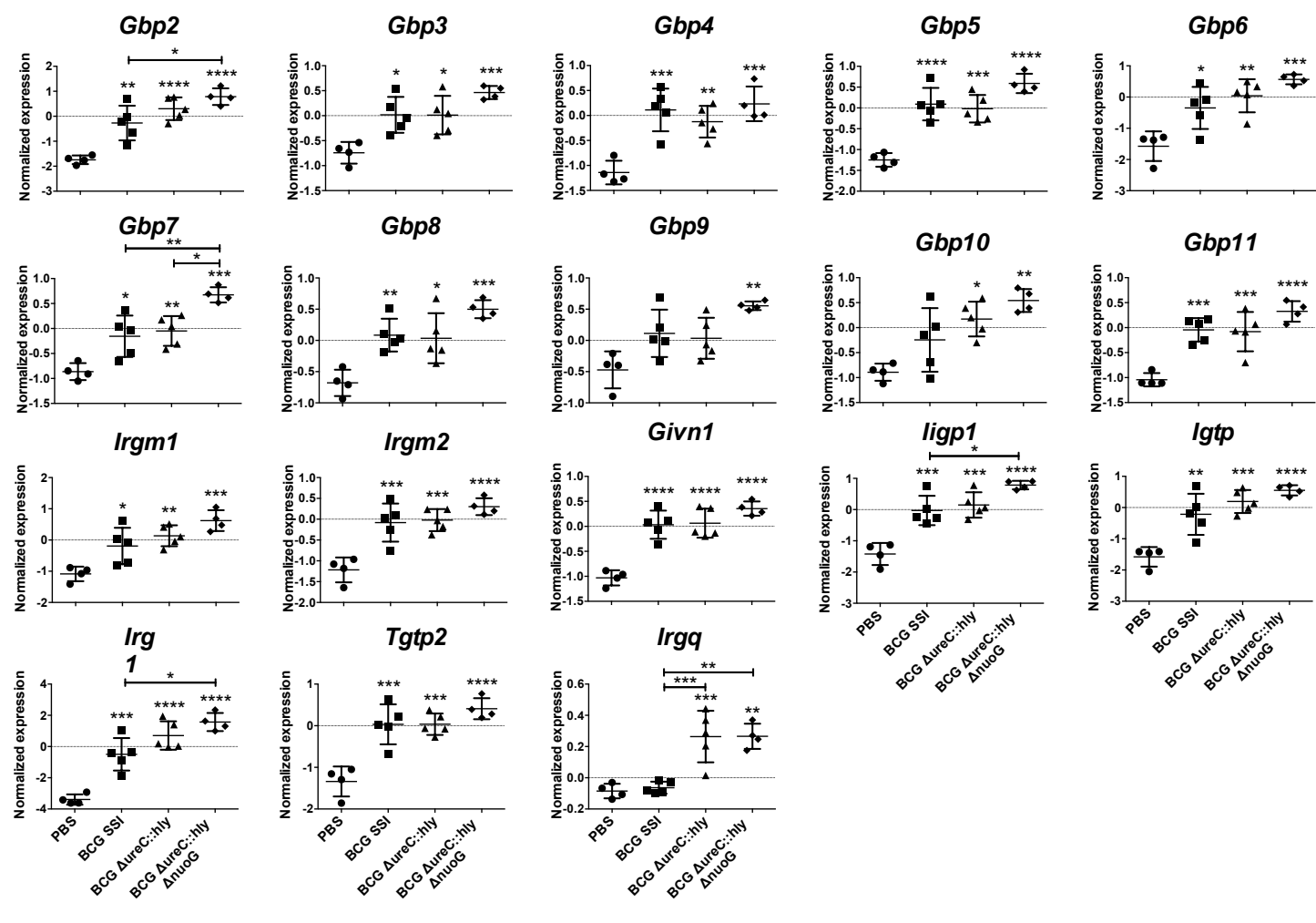

Supplement: Figure S4 — Differential expression of IFN-inducible GTPases 7 days after vaccination with BCG, BCG ΔureC::hly, and BCG ΔureC::hly ΔnuoG. Draining lymph nodes were collected at day 7 postvaccination, and gene expression was analyzed by whole-genome mouse microarray. Microarray data were analyzed using GeneSpring 12.6 GX (Agilent Technologies), with quality control filters, normalization, and one-way ANOVA. *, P < 0.05; **, P < 0.01; ***, P < 0.001; ****, P < 0.0001. PBS, phosphate-buffered saline. Download [file mbo003162827sf4.pdf]

***Il1b***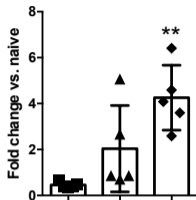***Il18***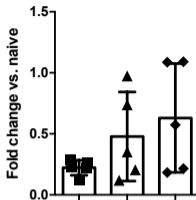***Ifi204***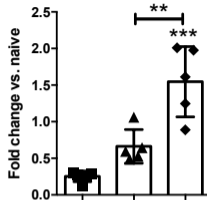***Gbp2***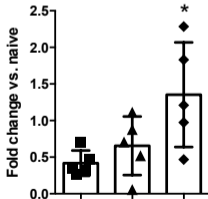***Gbp3***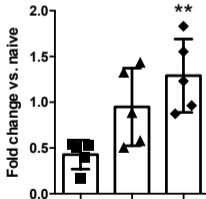***Gbp5***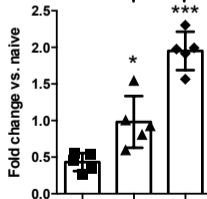

■ BCG    ▲ BCG  $\Delta ureC::hly$     ♦ BCG  $\Delta ureC::hly \Delta nuoG$

Supplement: Figure S5 — Upregulation of inflammasome-associated genes IL-1β, IL-18, and Ifi204 and Gbps at day 3 was validated by RT-PCR. RNA from lymph nodes of mice vaccinated with BCG, BCG ΔureC::hly, and BCG ΔureC::hly ΔnuoG was reverse transcribed to cDNA, and PCRs were performed on a Step One Plus real-time PCR machine. Analysis was performed using the comparative CT method with Ywhaz used as a housekeeping gene, and values from lymph nodes of vaccinated groups (n = 5) were compared to the average value of the naive control group (n = 5). Results are shown as fold difference compared to the average value of the naive control group. Significant differences are indicated by asterisks (*, P < 0.05; **, P < 0.01; ***, P < 0.001) in comparison to the BCG-vaccinated group, unless otherwise indicated by brackets. Download [file mbo003162827sf5.pdf]
