# Supplementary material for: Deletion of nuoG from the Vaccine Candidate Mycobacterium bovis BCG ΔureC::hly Improves Protection against Tuberculosis
Source: mBio. 2016 May 24;7(3):e00679-16. doi: 10.1128/mBio.00679-16 (PMC4895111; doi:10.1128/mBio.00679-16)
Supplement: Table S1 — Lists of genes in the heat map gene clusters labeled R1 to R8. A heat map was generated in GeneSpring from genes significantly differentially regulated (P < 0.05) compared to naive controls, and lists were compiled of gene clusters. Long noncoding RNAs are not included. Confirmed or potential gene/protein functions were obtained from the Mouse Gene Detail (MGI) (http://www.informatics.jax.org), GeneCards (http://www.genecards.org/), National Center for Biotechnology Information (NCBI) (http://www.ncbi.nlm.nih.gov/), and UniProt (http://www.uniprot.org) online repositories. [file mbo003162827st1.docx]

| **Table S1** Lists of genes^a^ in the heatmap gene clusters labeled R1-R8. | | | |
| --- | --- | --- | --- |
|  | |  |  |
| **CLUSTER R1** | |  |  |
| **GENE SYMBOL** | | **DESCRIPTION** | **KNOWN OR PREDICTED FUNCTION(S)^b^** |
| Il21 | | Interleukin 21 | Plays a role in both innate and adaptive immune responses by inducing the differentiation, proliferation and activity of multiple target cells including macrophages, natural killer cells, B cells and cytotoxic T cells. Required for optimal antibody production. Stimulates IFN-γ production in T-cells and NK cells in synergy with IL-18 and IL-15. |
| Bcat1 | | Branched chain aminotransferase 1, cytosolic | Catalyzes the reversible transamination of branched-chain alpha-keto acids to branched-chain L-amino acids essential for cell growth. |
| Esco2 | | Establishment of cohesion 1 homolog 2 (*S. cerevisiae*) | May have acetyltransferase activity and may be required for the establishment of sister chromatid cohesion during the S phase of mitosis. |
| Plk4 | | Polo-like kinase 4 | Regulates centriole duplication during the cell cycle. |
| Brip1 | | BRCA1 interacting protein C-terminal helicase 1 | Required for the maintenance of chromosomal stability. Involved in the repair of DNA double-strand breaks by homologous recombination. |
| Ect2 | | Ect2 oncogene | Cell cycle, DNA synthesis. |
| Cdc45 | | Cell division cycle 45 | Initiation of DNA replication. |
| Cdc25c | | Cell division cycle 25C | Regulation of cell division. |
| Mastl | | Microtubule associated serine/threonine kinase-like | Regulator of mitosis entry and maintenance. |
| I830127L07Rik | | Predicted: *Mus musculus* RIKEN cDNA I830127L07 gene | Predicted: Ly6-like protein. |
| Dlgap5 | | Discs, large (*Drosophila*) homolog-associated protein 5 | Mitotic phosphoprotein regulated by the ubiquitin-proteasome pathway. Key regulator of adherens junction integrity and differentiation. |
| Ska3 | | Spindle and kinetochore-associated complex subunit 3 | Component of the spindle and kinetochore-associated protein complex that regulates microtubule attachment to the kinetochores during mitosis. |
| Clspn | | Claspin | Triggers a checkpoint arrest of the cell cycle in response to replicative stress or DNA damage. |
| Fam83d | | Family with sequence similarity 83, member D | Required for proper chromosome congression and alignment during mitosis. |
| Tyms-ps | | Thymidylate synthase, pseudogene | Pseudogene. |
| Rps8 | | Ribosomal protein S8 | Structural constituent of ribosome. |
| Nebl | | Nebulette | Binds to actin and plays an important role in the assembly of the Z-disk. Isoform 2 might play a role in the assembly of focal adhesion. |
| Asns | | Asparagine synthetase | Metabolism. Synthesis of asparagine. |
| Gcat | | Glycine C-acetyltransferase (2-amino-3-ketobutyrate-coenzyme A ligase) | Metabolism. Catalyzes the reaction between 2-amino-3-ketobutyrate and coenzyme A to form glycine and acetyl-CoA. |
| Psat1 | | Phosphoserine aminotransferase 1 | Metabolism. Catalyzes the reversible conversion of 3-phosphohydroxypyruvate to phosphoserine and of 3-hydroxy-2-oxo-4-phosphonooxybutanoate to phosphohydroxythreonine. |
| Mfsd2a | | Major facilitator superfamily domain containing 2A | Transports lysophosphatidylcholine carrying long-chain fatty acids. |
| Mcm5 | | Minichromosome maintenance deficient 5, cell division cycle 46 (*S. cerevisiae*) | Involved in the initiation of DNA replication. |
| Kif2c | | Kinesin family member 2C | Regulates the turnover of microtubules at the kinetochore and functions in chromosome segregation during mitosis. |
| 2810408I11Rik | | RIKEN cDNA 2810408I11 gene | Unknown. |
| Rmi2 | | RMI2, RecQ-mediated genome instability 2, homolog (*S. cerevisiae*) | Plays a role in homologous recombination-dependent DNA repair. |
| Chaf1a | | Chromatin assembly factor 1, subunit A (p150) | Core component of a complex thought to mediate chromatin assembly in DNA replication and DNA repair. |
| Gins2 | | GINS complex subunit 2 (Psf2 homolog) | Part of a complex that plays an essential role in the initiation of DNA replication, and progression of DNA replication forks. It seems to bind preferentially to single-stranded DNA. |
| Tmem97 | | Transmembrane protein 97 | Plays a role in controlling cellular cholesterol levels. |
| Foxm1 | | Forkhead box M1 | Transcriptional factor regulating the expression of cell cycle genes essential for DNA replication and mitosis. Plays a role in the control of cell proliferation. Also plays a role in DNA break repair, participating in the DNA damage checkpoint response. |
| Tpx2 | | TPX2, microtubule-associated protein homolog (*Xenopus laevis*) | Required for normal assembly of mitotic spindles. Required for normal assembly of microtubules during apoptosis. Required for chromatin and/or kinetochore dependent microtubule nucleation. |
| Cdc6 | | Cell division cycle 6 | Functions as regulator during the early steps of DNA replication |
| Kntc1 | | Kinetochore associated 1 | Involved in mechanisms to ensure proper chromosome segregation during cell division. |
| Mybl2 | | Myeloblastosis oncogene-like 2 | Involved in cell cycle progression, and activation of the cell division cycle 2, cyclin D1, and insulin-like growth factor-binding protein 5 genes. |
| Cks2 | | CDC28 protein kinase regulatory subunit 2 | Cell cycle. Binds to the catalytic subunit of the cyclin-dependent kinases and is essential for their biological function. |
| Exo1 | | Exonuclease 1 | Functions in DNA mismatch repair. Required for somatic hypermutation and class switch recombination of immunoglobulin genes |
| Rad51 | | RAD51 homolog | Participates in a common DNA damage response pathway associated with the activation of homologous recombination and double-strand break repair. |
|  | |  |  |
| **CLUSTER R2** | |  |  |
| **GENE SYMBOL** | | **DESCRIPTION** | **KNOWN OR SUSPECTED FUNCTION(S)** |
| Lrr1 | | Leucine rich repeat protein 1 | Negative regulator of TNFRSF9-mediated signaling cascades. Negative regulator of signalling cascades which result in the activation of NK-kappaB and JNK1. |
| Gzmb | | Granzyme B | Necessary for target cell lysis in cell-mediated immune responses. Cleaves caspase-3, -7, -9 and 10 to give rise to active enzymes mediating apoptosis. |
| Soat2 | | Sterol O-acyltransferase 2 | Cholesterol metabolism. Plays a role in lipoprotein assembly and dietary cholesterol absorption. |
| Plac8 | | Placenta-specific 8 | Antimicrobial effector molecule, increases ability to kill ingested bacteria. Suggested to be similar to granzyme. |
| Serpina3g | | Serine (or cysteine) peptidase inhibitor, clade A, member 3G | May facilitate the survival of progenitor T-cells by protecting against apoptosis by inhibition of cathepsin B released from the lysosome, leading to the subsequent development of long-term memory CD8^+^ T-cells. |
| Gm12250 | | *Mus musculus* predicted gene 12250 | Unknown. |
| Ffar2 | | Free fatty acid receptor 2 | Activated by short chain fatty acids, plays a role in the regulation of whole-body energy homeostasis and in immunity. Activation by extracellular lectins may lead to activation of monocytes and induce the secretion of IL-8 in response to the presence of microbes. |
| Batf2 | | Basic leucine zipper transcription factor, ATF-like 2 | AP-1 family transcription factor that controls the differentiation of lineage-specific cells in the immune system. |
| Klra2 | | Killer cell lectin-like receptor, subfamily A, member 2 (Ly49b) | Part of natural killer complex, recognizes MHC-I and MHC-I-like proteins. |
| Cxcl10 | | (C-X-C motif) ligand 10 | Antimicrobial, stimulation of monocytes, natural killer and T-cell migration, and modulation of adhesion molecule expression. |
| Gpr141 | | G protein-coupled receptor 141 | Associated with respiratory and immune disorders. |
| Zbp1 | | Z-DNA binding protein 1 | Binds to foreign DNA and induces type-I IFN production. |
| Gm15056 | | Predicted gene 15056 | Defensin gene cluster. |
| Ly6g | | Lymphocyte antigen 6 complex, locus G | Neutrophil recruitment, associates with integrins, adhesion. |
| Gm10238 | | Predicted pseudogene 10238 | - |
| 9030619P08Rik | | RIKEN cDNA 9030619P08 gene | Lymphocyte antigen 6 complex pseudogene. |
| Ly6a | | Lymphocyte antigen 6 complex, locus A | Possibly adhesion. |
| Ly6c1 | | Lymphocyte antigen 6 complex, locus C1 | Possibly adhesion. |
| Il12rb1 | | Interleukin 12 receptor, beta 1 | IL-12 receptor, promotes Th1 responses. |
| AA467197 | | Expressed sequence AA467197 | Mir-147: induced upon TLR stimulation regulates murine macrophage inflammatory responses. |
| Igtp | | Interferon gamma induced GTPase | Involved in phagosomal maturation and cross-presentation, controls accumulation of lipid bodies, may disrupt vacuoles. |
| Gbp2 | | Guanylate binding protein 2 | IFN-inducible, immunity, intracellular resistance, associates with autophagy protein LC3. |
| Il6 | | Interleukin 6 | Moderates the acute phase immune response, promotes B cell growth and production of neutrophils |
| F10 | | Coagulation factor X | Converts prothrombin to thrombin in the presence of factor Va, calcium and phospholipid during blood clotting. |
| Slc16a3 | | Solute carrier family 16 (monocarboxylic acid transporters), member 3 (Monocarboxylic Acid Transporter 4) | Metabolism. Required to maintain a high rate of glycolysis in macrophages that is essential for a fully activated immune response. Catalyzes the rapid transport across the plasma membrane of many monocarboxylates such as lactate, pyruvate, branched-chain oxo acids derived from leucine, valine and isoleucine, and the ketone bodies acetoacetate, beta-hydroxybutyrate and acetate. |
| Mapk13 | | Mitogen-activated protein kinase 13 | Proliferation, differentiation, transcription regulation and development. |
| Spint1 | | Serine protease inhibitor, Kunitz type 1 | Thought to be involved in the regulation of the proteolytic activation of hepatocyte growth factor in injured tissues. |
| Gm14005 | | Predicted gene 14005 | Unclassified non-coding RNA gene. |
| Nme1 | | NME/NM23 nucleoside diphosphate kinase 1 | Major role in the synthesis of nucleoside triphosphates other than ATP. Cell proliferation, differentiation and development, signal transduction, G protein-coupled receptor endocytosis, and gene expression. |
| Srm | | Spermidine synthase | Biosynthesis of polyamines, which mediate growth and differentiation. Arginine and proline metabolism. |
| Ppa1 | | Pyrophosphatase (inorganic) 1 | Phosphate metabolism of cells. |
|  | |  |  |
| **CLUSTER R3** | |  |  |
| **GENE SYMBOL** | | **DESCRIPTION** | **KNOWN OR SUSPECTED FUNCTION(S)** |
| Gria4 | | Glutamate receptor, ionotropic, AMPA4 (alpha 4) | Receptor for glutamate that functions as ligand-gated ion channel in the central nervous system and plays an important role in excitatory synaptic transmission. Glutamate receptors are also found on immune cells. Different expression on resting and activated T cells. |
| Igkv6-20 | | Ig kappa active mRNA from hybridoma 17-1A. Chain | Antibody. |
| Igkv6-29 | | Immunoglobulin kappa chain variable 6-29 | Antibody. |
| Sdf2l1 | | Stromal cell-derived factor 2-Like 1 | Unknown. Gene ontology: regulation of apoptosis, response to ER stress. |
| Isg20 | | Interferon-stimulated exonuclease (Isg20) | Acts on single-stranded RNA and has minor activity towards single-stranded DNA. |
| Tg | | Thyroglobulin | Substrate for the synthesis of thyroxine and triiodothyronine as well as the storage of the inactive forms of thyroid hormone and iodine. |
| Igkv3-2 | | Immunoglobulin kappa variable 3-2 | Antibody. |
| Igkv3-4 | | Immunoglobulin kappa variable 3-4 | Antibody. |
| Igkv3-1 | | Immunoglobulin kappa variable 3-1 | Antibody. |
| Igkv3-7 | | Immunoglobulin kappa variable 3-7 | Antibody. |
| Igkv5-39 | | Immunoglobulin kappa variable 5-39 | Antibody. |
| Igkv2-109 | | Immunoglobulin kappa variable 2-109 | Antibody. |
| Igkv2-137 | | Immunoglobulin kappa chain variable 2-137 | Antibody. |
| Gm20730 | | Predicted gene, 20730 | Unknown. |
| Ighv1-42 | | Immunoglobulin heavy variable V1-42 | Antibody. |
| Chac1 | | ChaC, cation transport regulator 1 | Pro-apoptotic. Negative regulator of Notch signalling. Catalyzes the cleavage of glutathione into 5-oxoproline and a Cys-Gly dipeptide. Glutathione depletion is an important factor for apoptosis initiation and execution. |
| Lman1 | | Lectin, mannose-binding, 1 | Mannose-specific lectin. May recognize sugar residues of glycoproteins, glycolipids, or glycosylphosphatidyl inositol anchors and may be involved in the sorting or recycling of proteins, lipids, or both. |
| Ada | | Adenosine deaminase | Hydrolysis of adenosine to inosine (purine metabolism). Deficiency in this enzyme causes a form of severe combined immunodeficiency disease, in which there is dysfunction of both B and T lymphocytes with impaired cellular immunity and decreased production of immunoglobulins. |
| Creld2 | | Cysteine-rich with EGF-like domains 2 | Unknown. |
| Ergic1 | | Endoplasmic reticulum-Golgi intermediate compartment | A cycling membrane protein which is an ER-Golgi intermediate compartment protein which interacts with other members of this protein family to increase their turnover. |
| Slpi | | Secretory leukocyte peptidase inhibitor | Secreted inhibitor which protects epithelial tissues from attack by endogenous proteolytic enzymes. This antimicrobial protein has antibacterial, antifungal and antiviral activity. |
| Nmral1 | | NmrA-like family domain containing 1 | NADPH sensor protein (Redox sensor protein) that preferentially binds to NADPH. Negatively regulates the activity of NF-kappaB in an ubiquitination-dependent manner. Plays a key role in antiviral response by negatively regulating the IFN response factor 3-mediated expression of IFN-β. |
| Igkv1-110 | | Immunoglobulin kappa variable 1-110 | Antibody. |
| Igkv2-116 | | Immunoglobulin kappa variable 2-116 | Antibody. |
| Igkv1-117 | | Immunoglobulin kappa variable 1-117 | Antibody. |
| Igkv2-116 | | Immunoglobulin kappa variable 2-116 | Antibody. |
| Igkv1-132 | | Immunoglobulin kappa variable 1-132 | Antibody. |
| Igkv1-133 | | Immunoglobulin kappa variable 1-133 | Antibody. |
| Igkv1-122 | | Immunoglobulin kappa chain variable 1-122 | Antibody. |
| Igh | | Immunoglobulin VDJ region (A8H). | Antibody. |
| Igkv4-57-1 | | Immunoglobulin kappa variable 4-57-1 | Antibody. |
| Igkv4-80 | | Immunoglobulin kappa variable 4-80 | Antibody. |
| IGKV13-85 | | Anti-DNA antibody Ig kappa chain mRNA, V-J region, hybridoma 52.45, partial cds. | Antibody. |
| Mzb1 | | Marginal zone B and B1 cell-specific protein 1 | Promotes IgM assembly and secretion. Isoform 2 may be involved in regulation of apoptosis. Helps to diversify peripheral B-cell functions by regulating Ca(2+) stores, antibody secretion and integrin activation. Acts as a hormone-regulated adipokine/proinflammatory cytokine that is implicated in causing chronic inflammation, affecting cellular expansion and blunting insulin response in adipocytes. May have a role in the onset of insulin resistance. |
| Derl3 | | Der1-like domain family, member 3 | Appears to be involved in the degradation of misfolded glycoproteins in the ER. |
|  | |  |  |
| **CLUSTER R4** | |  |  |
| **GENE SYMBOL** | | **DESCRIPTION** | **KNOWN OR SUSPECTED FUNCTION(S)** |
| Fabp5 | | Fatty Acid Binding Protein 5 | Fatty acid uptake, transport, and metabolism. Polymorphisms in this gene are associated with type 2 diabetes. |
| Fpr1 | | Formyl Peptide Receptor 1 | Antibacterial host defence and inflammation. High affinity receptor for N-formyl-methionyl peptides, which are powerful neutrophil chemotactic factors. Activation of formyl-peptide receptors (FPRs) mediates induction of neutrophil chemotaxis, production of reactive oxygen species (ROS) and stimulation of degranulation of neutrophils. In addition, FPRs have a role in neutrophil transcriptional regulation and cytokine production, and induce neutrophil apoptosis in a ROS-dependent manner. |
| Gm5150 | | Predicted gene | Unknown. |
| LOCI02642410 | | Tyrosine-protein phosphatase non-receptor type substrate 1-like | Unknown. |
| Chil3 | | Chitinase-like 3 (YKL-40) | Inflammation and tissue remodeling. Secreted by macrophages and neutrophils. Linked to activation of the AKT pro-survival (anti-apoptotic) signaling pathway. |
| Slc13a3 | | Solute carrier family 13 (sodium-dependent dicarboxylate transporter), member 3 | Transports succinate and other Krebs cycle intermediates. |
| Clec4a1 | | C-type lectin domain family 4, member a1 (mDcir4 -Dendritic cell inhibitory receptor 4) | Pattern recognition receptor. |
| FcgrI | | Fc receptor, IgG, high affinity I | Antibody dependent killing, antigen presentation, inflammatory responses, immune complex formation. |
| Wfdc17 | | WAP four-disulfide core domain 17 | Unknown. |
| Ms4a4a | | Membrane-spanning 4-domains, subfamily A, member 4A | May be involved in signal transduction as a component of a multimeric receptor complex. |
| Saa3 | | Serum amyloid A 3 | Secreted in acute phase of inflammation, recruitment of inflammatory cells, induction of enzymes that degrade extracellular matrix, cholesterol transport. |
| Nxpe5 | | Neurexophilin and pc-esterase domain family, member 5 | Unknown. |
| Pydc4 | | Pyrin domain containing 4 | Possible negative regulation of transcription from RNA polymerase II promoter. |
| Mmp3 | | Matrix metallopeptidase 3 | Degrades fibronectin, laminin, collagens III, IV, IX, and X, and cartilage proteoglycans. |
| Oas2 | | 2'-5'-oligoadenylate synthetase 2 | Interferon-induced, dsRNA-activated antiviral enzyme which plays a critical role in cellular innate antiviral responses, apoptosis, cell growth, differentiation and gene regulation. |
| Rtp4 | | Receptor (chemosensory) transporter Protein 4 | Unknown. IFN-induced. |
| AW011738 | | Expressed sequence | Unknown. |
| Lcn2 | | Lipocalin 2 / neutrophil gelatinase-associated lipocalin (NGAL) | Involved in innate immunity by sequestrating iron to limit bacterial growth. Expressed in neutrophils. |
| Slc7a5 | | Solute carrier family 7 (amino acid transporter light chain, L system), member 5 | Promotes metabolic reprogramming necessary for T cell differentiation by sustaining cMyc expression. Sodium-independent, high-affinity transport of large neutral amino acids. Involved in cellular amino acid uptake. |
| Hp | | Haptoglobin | Binds free plasma haemoglobin, which allows degradative enzymes to gain access to the haemoglobin, while at the same time preventing loss of iron through the kidneys and protecting the kidneys from damage by haemoglobin. |
| Ifitm6 | | Interferon-induced transmembrane protein 6 | Immunity. IFITM proteins inhibit fusion of viral membrane with cellular endosomal or lysosomal vesicles by modifying lipid components or fluidity. |
| Slfn4 | | Schlafen 4 | Upregulated by type I IFNs and TLRs. Upregulated during macrophage activation and down-regulated during differentiation. Regulation of growth, modulates myelopoiesis. Reduced infiltration of monocytes and inflammatory macs in schlafen4 overexpressing mice. Splenomegaly in overexpressing mice. |
| Sct | | Secretin | May be an apoptosis inhibitor. Regulates bicarbonate levels. |
| Timp1 | | TIMP metallopeptidase inhibitor 1 | TIMPS are natural inhibitors of the matrix metalloproteinases. Promotes cell proliferation in a wide range of cell types, and may also have anti-apoptotic functions. |
| Oas3 | | 2'-5'-oligoadenylate synthetase 3 | Induced by IFNs and catalyzes the 2', 5' oligomers of adenosine in order to bind and activate RNase L. Plays a role in the inhibition of cellular protein synthesis and viral infection resistance, apoptosis, cell growth, differentiation and gene regulation. |
| Isg15 | | ISG15 ubiquitin-like modifier | Ubiquitin-like protein that is conjugated to intracellular target proteins upon activation by type 1 IFNs, chemotactic activity towards neutrophils, direction of ligated target proteins to intermediate filaments, cell-to-cell signaling, and antiviral activity. |
| Oasl2 | | 2'-5' oligoadenylate synthetase-like 2 | IFN-regulated/-induced. |
| Vcan | | Versican | Involved in cell adhesion, proliferation, proliferation, migration and angiogenesis and plays a central role in tissue morphogenesis and maintenance. |
| Oas1a | | 2'-5' oligoadenylate synthetase 1A | Type I IFN-induced, antiviral response, apoptosis, cell growth, differentiation and gene regulation. |
| Ifi204 | | Interferon activated gene 204 | Co-operates with cGAS to sense dsDNA and activate the STING-dependent type I IFN pathway. |
| Oas1f | | 2'-5' oligoadenylate synthetase 1F | Type I IFN-induced, antiviral response, apoptosis, cell growth, differentiation and gene regulation. |
| Spon1 | | Spondin 1, extracellular matrix protein | Cell adhesion protein. |
| MS4a4a | | Membrane-spanning 4-domains, subfamily A, member 4A (CD20 antigen-like 1) | Unknown. |
| Fcgr4 | | Fc receptor, IgG, low affinity IV | Serves as an activating receptor for IgG2a and IgG2b. Low-affinity IgE receptor for all IgE allotypes, ligation of Fc gamma receptor 4 by antigen-IgE immune complexes, promotes macrophage-mediated phagocytosis, presentation of antigen to T cells, production of proinflammatory cytokines and the late phase of cutaneous allergic reactions. |
| Sirpb1b | | Signal-regulatory protein beta 1 | Negative regulation of receptor tyrosine kinase-coupled signaling processes, may regulate phagocytosis. |
| Gm9733 | | Predicted gene | Unknown. |
| Sirpb1a | | Signal-regulatory protein beta 1A | Negative regulation of receptor tyrosine kinase-coupled signaling processes, may regulate phagocytosis. |
| Cxcr1 | | Chemokine (C-X-C motif) receptor 1 (interleukin 8 receptor, alpha) | Binds to IL8 with high affinity, and transduces the signal through a G-protein activated second messenger system, in Akt signaling pathway. |
| Ocstamp | | Osteoclast stimulatory transmembrane protein | Plays a role in cellular fusion and cell differentiation. Assists in modulating cell-cell fusion in both osteoclasts and foreign body giant cells. |
| Socs3 | | Suppressor of cytokine signaling 3 | Negative regulators of cytokine signaling, inhibits the activity of JAK2 kinase. |
| Ccl7 | | Chemokine (C-C motif) ligand 7, monocyte chemoattractant protein 3 | A secreted chemokine which attracts macrophages during inflammation and metastasis. |
| Htr7 | | 5-hydroxytryptamine (serotonin) receptor 7, adenylate cyclase-coupled | Serotonin receptor. |
| Prm1 | | Protamine 1 | Protamines substitute for histones in the chromatin during the haploid phase of spermatogenesis. They compact DNA into a highly condensed, stable and inactive complex. |
| Gdf3 | | Growth differentiation factor 3 | Member of the bone morphogenetic protein family and the TGF-beta superfamily. An important paralog of this gene is BMP2. Regulators of cell growth and differentiation. Can inhibit apoptosis. |
| Ccl2 | | Chemokine (C-C motif) ligand 2 | Chemotactic activity for monocytes and basophils. Implicated in the pathogenesis of diseases characterized by monocytic infiltrates. |
|  | |  |  |
| **CLUSTER R5** | |  |  |
| **GENE SYMBOL** | | **DESCRIPTION** | **KNOWN OR SUSPECTED FUNCTION(S)** |
| Htr7 | | 5-hydroxytryptamine (serotonin) receptor 7 | Serotonin receptor, regulation of circadian rhythms, smooth muscle relaxation, learning and memory. |
| Prm1 | | Protamine 1 | Protamines substitute for histones in the chromatin during the haploid phase of spermatogenesis. They compact DNA into a highly condensed, stable and inactive complex. |
| Gdf3 | | Growth differentiation factor 3 | Member of the bone morphogenetic protein family and the TGF-β superfamily, regulators of cell growth and differentiation. |
| Ccl2 | | Chemokine (C-C motif) ligand 2 (MCP-1) | Chemotactic activity for monocytes and basophils, implicated in the pathogenesis of diseases characterized by monocytic infiltrates. |
| Bex6 | | Brain expressed gene 6 | Transcription elongation factor family. |
| Gm9706 | | *Mus musculus* predicted gene 9706 | Unknown |
| Cxcl1 | | Chemokine (C-X-C motif) ligand 1 | Plays a role in inflammation and as a chemoattractant for neutrophils. |
| Gpr84 | | G protein-coupled receptor 84 | Receptor for medium-chain free fatty acid with carbon chain lengths of C9 to C14. May have important roles in processes from fatty acid metabolism to regulation of the immune system. |
| Il1b | | Interleukin 1 beta | An important mediator of the inflammatory response, and is involved in a variety of cellular activities, including cell proliferation, differentiation, and apoptosis. |
| Pla2g7 | | Phospholipase A2, group VII (platelet-activating factor acetylhydrolase, plasma) | Catalyzes the degradation of platelet-activating factor. |
| Fpr2 | | Formyl peptide receptor 2 | FPRs are involved in antibacterial host defence and inflammation. Activation of FPRs mediates induction of neutrophil chemotaxis, production ROS to clear damaged cells and stimulation of degranulation of neutrophils. In addition, FPRs have a role in neutrophil transcriptional regulation and cytokine production, and induce neutrophil apoptosis in a ROS-dependent manner. |
| Dbx2 | | Developing brain homeobox 2 | Homeobox family of transcription factors, many of which are involved in developmental processes, may play a role in hematopoietic differentiation. |
| Fabp7 | | Fatty acid-binding protein 7, brain | Roles in fatty acid uptake, transport, and metabolism. |
| Tnfaip6 | | Tumor necrosis factor alpha-induced protein 6 | Involved in extracellular matrix stability and cell migration, can be induced by proinflammatory cytokines such as TNF-α and IL-1, possibly involved in cell–cell and cell–matrix interactions during inflammation and tumorigenesis. |
| Tnn | | Tenascin N | Extracellular matrix glyocoprotein, integrin binding, PI3K-Akt signaling pathway. |
| Chl1 | | Cell adhesion molecule with homology to L1CAM | Cell adhesion, potentiates integrin-dependent cell migration towards extracellular matrix proteins |
| Selp | | Selectin, platelet | Ca(2+)-dependent receptor for myeloid cells (CAM) that binds to carbohydrates on neutrophils and monocytes. Mediates the interaction of activated endothelial cells or platelets with leukocytes. Mediates rapid rolling of leukocytes over vascular surfaces during the initial steps in inflammation. CAMs have roles in cell proliferation, differentiation, motility, trafficking, apoptosis and tissue architecture. |
| Retnlg | | Resistin like gamma | Innate immune-derived proinflammatory cytokine, expressed in macrophages. Associated with insulin resistance. |
| Il4 | | Interleukin 4 | Th2, PI3K-Akt signaling pathway, optimal Th1 responses, humoral responses, B cell activation. |
| Chil1 | | Chitinase-like 1 | Promotes Th2 responses, alternatively activated macrophages and DC recruitment. Inhibits apoptosis. |
| 1100001G20Rik (Wfdc21) | | WAP four-disulfide core domain 21 | Response to LPS, metalloenzyme activator. |
| S100a9 | | S100 calcium binding protein A9 (calgranulin B) | Antimicrobial protein exhibiting antifungal and antibacterial activity. Regulation of cellular processes such as cell cycle progression and differentiation, inflammatory processes and immune responses. Can induce neutrophil chemotaxis, adhesion, and increases the bactericidal activity of neutrophils by promoting phagocytosis. Can induce degranulation of neutrophils. Proinflammatory, oxidant-scavenging and apoptosis-inducing activities. Can induce cell death via autophagy and apoptosis which occurs through the crosstalk of mitochondria and lysosomes via ROS. |
| S100a8 | | S100 calcium binding protein A8 (calgranulin A) | Plays a prominent role in the regulation of inflammatory processes and immune response. It can induce neutrophil chemotaxis and adhesion. Predominantly found as calprotectin (S100A8/A9). Can regulate neutrophil number and apoptosis by an anti-apoptotic effect; Can induce cell death via autophagy and apoptosis. |
| Eppk1 | | Epiplakin 1 | Play a role in the organization of cytoskeletal architecture. |
| Mcpt2 | | Mast cell protease 2 (Mcpt2) | Regulated by IL-3, IL-10 and IL-15. Virtually without enzymatic activity. May play a role in inflammatory response to bacteria. |
| Cd5l | | CD5 antigen-like | May play a role in the regulation of the immune system. Seems to play a role as an inhibitor of apoptosis |
| Saa1 | | Serum amyloid A 1 | A major acute phase protein that is highly expressed in response to inflammation and tissue injury. also plays an important role in HDL metabolism and cholesterol homeostasis |
| Ccl6 | | Chemokine (C-C motif) ligand 6 | Antibacterial peptide, promotes innate immunity, expressed in myeloid cells. |
|  | |  |  |
| **CLUSTER R6** | |  |  |
| **GENE SYMBOL** | | **DESCRIPTION** | **KNOWN OR SUSPECTED FUNCTION(S)** |
| Gm525 | | Predicted gene | Unknown. |
| Dmbt1 | | Surfactant pulmonary-associated D-binding protein /deleted in malignant brain tumors 1 | Defence against bacterial pathogens, broad bacterial binding specificity, possible opsonin receptor, potential tumour suppressor gene. |
| Krt7 | | keratin-7 | Structural, blocks interferon-dependent interphase and stimulates DNA synthesis in cells. |
| Cyp2e1 | | Cytochrome P450, family 2, subfamily E, polypeptide 1 | Enzyme, metabolizes both endogenous and exogenous substrates (e.g. ethanol, acetone, benzene, carbon tetrachloride, ethylene glycol, nitrosamines). |
| Fabp4 | | Fatty acid binding protein 4, adipocyte | Fatty acid uptake, transport, and metabolism. |
| Slc2a4 | | Solute carrier family 2 (facilitated glucose transporter), member 4 | Insulin-regulated facilitative glucose transporter. |
| Plin4 | | Perilipin 4 | May play a role in triacylglycerol packaging into adipocytes. May function in the biogenesis of lipid droplets. |
| Me1 | | Malic enzyme 1, NADP(+)-dependent, cytosolic | Generates NADPH for fatty acid biosynthesis. |
| Thrsp | | Thyroid hormone responsive (SPOT14 homolog | Plays a role in the regulation of lipogenesis. Important for the biosynthesis of triglycerides with medium-length fatty acid chains. May function as transcriptional coactivator. May modulate the transcription factor activity of THRB. |
| Gpd1 | | Glycerol-3-phosphate dehydrogenase 1 (soluble) | Carbohydrate and lipid metabolism, form a glycerol phosphate shuttle that facilitates the transfer of reducing equivalents from the cytosol to mitochondria. |
| Mup20 | | Major urinary protein 20, darcin | [Pheremone binding, insulin-activated receptor activity, cellular response to lipid, regulation of lipid and glucose metabolism and insulin secretion](http://www.ebi.ac.uk/QuickGO/GTerm?id=GO:0005009). |
| Car3 | | Carbonic anhydrase 3 | Catalyzes carbon dioxide, responds to oxidative stress, down-regulated by insulin, increased expression in obesity. |
| Adig | | Adipogenin | Adipocyte differentiation. |
| Adipoq | | Adiponectin, C1Q and collagen domain containing | Control of fat metabolism and insulin sensitivity, with direct anti-diabetic, anti-atherogenic and anti-inflammatory activities, antagonism of TNF-α by negative regulation of its expression, inhibition of endothelial NF-kappa-B signaling through a cAMP-dependent pathway. May play a role in cell growth, angiogenesis and tissue remodeling by binding and sequestering various growth factors. |
| Mup2 | | Major urinary protein 2 | Pheremone binding, lipid biosynthesis, glucose homeostasis, insulin-activated receptor activity. |
| Cfd | | Complement factor D (Adipsin) | Component of alternative complement pathway, serine protease. |
| Retn | | Resistin | Hormone, potentially links obesity to type II diabetes, suppresses insulin ability to stimulate glucose uptake into adipose cells. |
| Mup-ps12 | | Major urinary protein, pseudogene 12 | Pseudogene. |
| Mup19 | | Major urinary protein 19 | Pheremone binding, lipid and glucose metabolism. |
| Mup17 | | Major urinary protein 17 | Pheremone binding, lipid and glucose metabolism. |
| Cited | | Cbp/P300-interacting transactivator, with Glu/Asp-rich carboxy-terminal domain, 1 | Transcriptional coactivator of the p300/CBP-mediated transcription complex, enhances SMAD-mediated transcription |
| Krt18 | | beratin 18, Type I | Structural, expressed in single layer epithelial tissue. |
| Gsta2 | | [Glutathione S-transferase alpha 2](http://www.genenames.org/cgi-bin/gene_symbol_report?hgnc_id=4627) | Detoxification of electrophilic compounds by conjugation with glutathione, including carcinogens, therapeutic drugs, environmental toxins and products of oxidative stress. |
| Postn | | Periostin, osteoblast specific factor | Induces cell attachment and spreading and plays a role in cell adhesion. |
| Cnn1 | | Calponin 1, basic, smooth muscle | Regulation and modulation of smooth muscle contraction. Binds to actin, calmodulin, troponin C and tropomyosin. |
| Ptn | | Pleiotrophin | Anti-apoptotic. Mitogenic for fibroblasts, epithelial, and endothelial cells. Binds anaplastic lymphoma kinase which induces MAPK pathway activation, an important step in anti-apoptotic signaling and regulation of cell proliferation. |
| CD209f | | DC-SIGN | pathogen recognition receptor, functions in innate immunity, mediates endocytosis of pathogens. |
|  | |  |  |
| **CLUSTER R7** | |  |  |
| **GENE SYMBOL** | | **DESCRIPTION** | **KNOWN OR SUSPECTED FUNCTION(S)** |
| A730062M13Rik | | 7 days neonate cerebellum cDNA, RIKEN full-length enriched library | Unknown. |
| Abca8a | | ATP-binding cassette, sub-family A (ABC1), member 8a | ABC proteins transport various molecules across extra- and intracellular membranes. Probable transporter which may play a role in macrophage lipid homeostasis (by similarity). |
| Mucl1 | | Mucin-like 1 | Related pathways: Biosynthesis of the N-glycan precursor (dolichol lipid-linked oligosaccharide), cholesterol absorption. |
| Igfbp5 | | Insulin-like growth factor binding protein 5 | Related pathways: biosynthesis of the N-glycan precursor (dolichol lipid-linked oligosaccharide), IGF-binding proteins prolong the half-life of the IGFs and have been shown to either inhibit or stimulate the growth promoting effects of the IGFs on cell culture. Downregulated by TGF-β. Family members in Wnt signaling network. |
| Fzd10 | | Frizzled homolog 10 (Drosophila) | Receptor for Wnt proteins. May be involved in transduction and intercellular transmission of polarity information during tissue morphogenesis and/or in differentiated tissues. |
| Cyp2f2 | | Cytochrome P450, family 2, subfamily f, polypeptide 2 | Detoxification. May be involved in the metabolism of various pneumotoxicants including naphthalene. The cytochrome P450 proteins are monooxygenases which catalyze many reactions involved in drug metabolism and synthesis of cholesterol, steroids and other lipids. |
| Fxyd2 | | FXYD domain-containing ion transport regulator 2 | May be involved in forming the receptor site for cardiac glycoside binding or may modulate the transport function of the sodium ATPase. Deficiency leads to increased glucose tolerance. |
| Wif1 | | Wnt inhibitory factor 1 | Binds to Wnt proteins and inhibits their activities. May be involved in mesoderm segmentation. Functions as a tumor suppressor gene. |
| Gm10790 | | Predicted gene 10790 | Non-coding RNA gene. |
| Cd209d | | CD209d antigen (DC-SIGN) | Pattern recognition receptor and cell adhesion receptor, innate immune system, recognizes *Mycobacteria*, may act as a DC rolling receptor, Seems to regulate DC-induced T-cell proliferation. |
| Atp8b5 | | ATPase, class I, type 8B, member 5 | Pseudogene. |
| Bpifb6 | | BPI fold-containing family B, member 6 | Bactericidal/permeability-increasing protein-like, lipid binding, innate immunity. |
| Bpifb3 | | BPI fold-containing family B, member 3 | May have the capacity to recognize and bind specific classes of odorants. May act as a carrier molecule, transporting odorants across the mucus layer to access receptor sites. May serve as a primary defence mechanism by recognizing and removing potentially harmful odorants or pathogenic microorganisms from the mucosa or clearing excess odorant from mucus to enable new odorant stimuli to be received. |
| Bpifb2 | | BPI fold-containing family B, member 2 | Lipid binding, encodes a member of the lipid transfer/lipopolysaccharide binding protein gene family. |
| Hamp2 | | Hepcidin antimicrobial peptide 2 | Antimicrobial peptide. |
| Syngr1 | | Synaptogyrin 1 | Integral membrane protein associated with presynaptic vesicles in neuronal cells, function unclear. |
| Syndig1l | | Synapse differentiation inducing 1 like | Interferon induced, function unknown. |
| Tle2 | | Transducin-like enhancer of split 2, homolog of Drosophila E (spl) | Transcriptional corepressor that binds to a number of transcription factors. Inhibits the transcriptional activation mediated by CTNNB1 and TCF family members in Wnt signalling. |
| Gdf10 | | Growth differentiation factor 10 (BMP3B) | Member of the bone morphogenetic protein family and the TGF-β superfamily regulators of cell growth, differentiation and activation. |
|  | |  |  |
| **CLUSTER RR8** |  | |  |
| **GENE SYMBOL** | | **DESCRIPTION** | **KNOWN OR SUSPECTED FUNCTION(S)** |
| Krt20 | | Keratin 20 | Structural protein. |
| Inmt | | Indolethylamine N-methyltransferase | Catalyzes the N-methylation of tryptamine and structurally related compounds. Possible role in the detoxification of selenium compounds (by similarity). |
| LOC102637409 | | Uncharacterized LOC102637409 | Unknown. |
| D130061D10Rik | | 12 days embryo spinal ganglion cDNA, RIKEN full-length enriched library | Unknown. |
| Srpx2 | | Sushi-repeat-containing protein, X-linked 2 | Cellular migration and adhesion, angiogenesis. Acts as a ligand for the urokinase plasminogen activator surface receptor. |
| Ryr3 | | Ryanodine receptor 3 | Functions to release calcium from intracellular storage for use in many cellular processes. |
| Pgm5 | | Phosphoglucomutase 5 | Component of adherens-type cell-cell and cell-matrix junctions. Lacks phosphoglucomutase activity. |
| Ano1 | | Anoctamin 1, calcium activated chloride channel | ANO1 functions as a calcium-activated chloride channel. It is ubiquitously expressed in epithelia and is thought to be important in epithelial fluid transport. |
| C1ql3 | | C1q-like 3 (C1q and tumor necrosis factor-related Protein 13 ) | Unknown. C1Q like proteins are ligands for the brain-specific angiogenesis inhibitor cell-adhesion G-protein coupled receptor family. May be involved in synapse homeostasis and DC morphology. |
| Zfp503 | | Zinc finger protein 503 | Unknown. |
| F8 | | Coagulation factor VIII | Participates in the intrinsic pathway of blood coagulation. |
| Chp2 | | Calcineurin-like EF hand protein 2 | A small calcium-binding protein that regulates cell pH by controlling plasma membrane-type Na+/H+ exchange activity, functions in the calcineurin/ nuclear factor of activated T cells signaling pathway, plays a role in the regulation of cell proliferation, protects cells from serum deprivation-induced death. |
| Ntng1 | | Netrin G1 | Involved in controlling patterning and neuronal circuit formation at the laminar, cellular, subcellular and synaptic levels. Promotes neurite outgrowth of both axons and dendrites. Pathway: cell adhesion molecules. |
| Ptgds | | Prostaglandin D2 synthase (brain) | Glutathione-independent prostaglandin D synthase that catalyzes the conversion of prostaglandin H2 to postaglandin D2 (PGD2). PGD2 functions as a neuromodulator as well as a trophic factor in the central nervous system. May have an anti-apoptotic role in oligodendrocytes. Binds small non-substrate lipophilic molecules, including retinoic acid and thyroid hormone, and may act as a scavenger for harmful hydrophobic molecules and as a secretory retinoid and thyroid hormone transporter. |
| Sult1a1 | | Sulfotransferase family 1A, phenol-preferring, member 1 | Sulfotransferase enzymes catalyze the sulfate conjugation of many hormones, neurotransmitters, drugs, and xenobiotic compounds. |
| Fam171a1 | | Family with sequence similarity 171, member A1 | Unknown. |
| Galnt15 | | UDP-N-acetyl-alpha-D-galactosamine:polypeptide N-acetylgalactosaminyltransferase 15 | Catalyzes the initial reaction in O-linked oligosaccharide biosynthesis, prefers Muc1a as substrate. |
| Dact1 | | Dapper homolog 1, antagonist of beta-catenin (xenopus) | Involved in regulation of intracellular signaling pathways during development. Specifically thought to play a role in canonical and/or non-canonical Wnt signaling pathways through interaction with Dishevelled family proteins. Activation/inhibition of Wnt signaling may depend on the phosphorylation status |
| Syt15 | | Synaptotagmin XV | Member of the Synaptotagmin family of membrane trafficking proteins. May be involved in the trafficking and exocytosis of secretory vesicles in non-neuronal tissues. |
| Kcna6 | | Potassium voltage-gated channel, shaker-related, subfamily, member 6 | Voltage-gated potassium channel that mediates transmembrane potassium transport in excitable membranes. |
| Nrxn1 | | Neurexin I | Cell surface protein involved in cell–cell interactions, exocytosis of secretory granules and regulation of signal transmission. Function is isoform-specific. |
| Prkg2 | | Protein kinase, cGMP-dependent, type II | Protein kinase G is a cyclic GMP-dependent protein serine/threonine kinase that phosphorylates a variety of biological targets. |
| Rxrg | | Retinoid X receptor gamma | Receptor for retinoic acid. RXRGs are common binding partners to many other nuclear receptors, including PPARs, liver X receptors and vitamin D receptors. |
| Ltbp4 | | Latent transforming growth factor beta binding protein 4 | Involved in the assembly, secretion and targeting of TGFβ1 to sites at which it is stored and/or activated. May play critical roles in controlling and directing the activity of TGFβ1. |
| Sned1 | | Sushi, nidogen and EGF-like domains 1 | Unknown. Insulin-responsive gene, paralog of Notch2, EGF-like domain (Notch family members play a role in a variety of developmental processes by controlling cell fate decisions). |
| Clec4g | | C-type lectin domain family 4, member g | A glycan-binding receptor and member of the C-type lectin family which plays a role in the T-cell immune response. |
| Creg2 | | Cellular repressor of E1A-stimulated genes 2 | Unknown. Oxidoreductase activity. |
| Il22ra2 | | Interleukin 22 receptor, alpha 2 | Binds to and inhibits IL-22 activity by blocking the interaction of IL-22 with its cell surface receptor, May play an important role as an IL-22 antagonist in the regulation of inflammatory responses, blocks Socs3 expression. |
| Cd209e | | CD209e antigen (DC-SIGN) | Pattern recognition receptor expressed on the surface of immature DCs and involved in initiation of primary immune response. |
| Il17rb | | Interleukin 17 receptor B | Receptor for the proinflammatory cytokines IL17B and IL17E. May play a role in controlling the growth and/or differentiation of hematopoietic cells. |

^a^ A heatmap was generated in GeneSpring from genes significantly differentially regulated (*p*<0.05) compared to naïve controls, and lists were compiled of gene clusters. Long non-coding RNAs are not included.

^b^ Confirmed or potential gene/protein functions were obtained from Mouse Gene Detail (MGI) (http://www.informatics.jax.org), GeneCards (http://www.genecards.org/), National Centre for Biotechnology information (NCBI) (http://www.ncbi.nlm.nih.gov/) and Uniprot (http://www.uniprot.org) online repositories.
