# Supplementary material for: Deletion of nuoG from the Vaccine Candidate Mycobacterium bovis BCG ΔureC::hly Improves Protection against Tuberculosis
Source: mBio. 2016 May 24;7(3):e00679-16. doi: 10.1128/mBio.00679-16 (PMC4895111; doi:10.1128/mBio.00679-16)
Supplement: Table S2 — Genes significantly upregulated (P < 0.05) at least 2-fold compared to naive control only in BCG ΔureC::hly ΔnuoG-vaccinated mice. Venn diagrams were plotted in GeneSpring to analyze overlap of genes significantly upregulated (P < 0.05) at least 2-fold compared to naive control in groups vaccinated with different strains of BCG, at 1, 3, and 7 days postvaccination. Lists of genes upregulated specifically in BCG ΔureC::hly ΔnuoG mice are given here with their potential or confirmed protein functions. Noncoding genes and confirmed pseudogenes were not included. Gene/protein functions were obtained from the Mouse Gene Detail (MGI) (http://www.informatics.jax.org), GeneCards (http://www.genecards.org/), National Center for Biotechnology Information (NCBI) (http://www.ncbi.nlm.nih.gov/), and UniProt (http://www.uniprot.org) online repositories. The P values corrected for multiple comparisons are shown. [file mbo003162827st2.doc]

**Table S2** Genes significantly upregulated (*p*<0.05) at least 2-fold compared to naïve control only in BCG Δ*ureC*::*hly* Δ*nuoG-*vaccinated mice^a^.

| **DAY 1** |  |  |  |  |
| --- | --- | --- | --- | --- |
| **GENE SYMBOL** | **FC** | **P(Corr)^b^** | **DESCRIPTION^c^** | **KNOWN OR SUSPECTED FUNCTION(S)** |
| **Dppa4** | 2.03 | 1.03E-04 | Developmental pluripotency associated 4 | Development, transcription, epigenetic modification. |
| **Il18** | 2.296 | 7.76E-04 | Interleukin-18 | Stimulates IFN-γ production in T-helper type I cells, augments natural killer cell activity. |
| **Slc7a5** | 2.39 | 1.26E-03 | Solute carrier family 7 (cationic amino acid transporter, y+ system), member 5 | High-affinity transport of large neutral amino acids. May be involved in peptide–antigen binding. |
| **Vmn2r7** | 2.376 | 1.55E-03 | Vomeronasal 2, receptor 7 | Pheremone receptor, lipid binding. |
| **Serpina3n** | 2.152 | 2.25E-03 | Serine (or cysteine) peptidase inhibitor, clade A, member 3N | Acute phase response, inflammatory response. Although its physiological function is unclear, it can inhibit neutrophil cathepsin G and mast cell chymase, both of which can convert angiotensin-1 to the active angiotensin-2. |
| **Cdc123** | 2.091 | 3.91E-03 | Cell division cycle 123 | Required for S phase entry of the cell cycle. |
| **Calml4** | 2.293 | 5.40E-03 | Calmodulin-like 4 | Belongs to family of sensor/signal transduction calcium binding proteins which are involved in many processes including inflammation, metabolism, apoptosis, intracellular movement, short-term and long-term memory, and the immune response. |
| **Cd209c** | 2.103 | 7.14E-03 | CD209c antigen (DC-SIGN) | Pattern-recognition receptor on DCs involved in initiation of primary immune response. Marker of inflammatory, monocyte-derived DCs. |
| **Itih4** | 2.101 | 2.53E-02 | Inter alpha-trypsin inhibitor, heavy chain 4 | Type II acute-phase protein involved in inflammatory responses to trauma. |
| **Fcer1a** | 2.069 | 3.05E-02 | Fc receptor, IgE, high affinity I, alpha polypeptide | IgE binding, expressed on mast cells, basophils and inflammatory DCs, role in allergic response. |
| **Serpinb7** | 2.929 | 4.34E-02 | Serine (or cysteine) peptidase inhibitor, clade B, member 7 | Serpins play a role in coagulation and inflammation. Protease inhibitor. Might function as an inhibitor of Lys-specific proteases. Might influence the maturation of megakaryocytes. |
| **Avpr1a** | 3.624 | 4.88E-02 | Arginine vasopressin receptor 1A | A receptor mediating cell contraction and proliferation, platelet aggregation, release of coagulation factor and glycogenolysis. |
|  |  |  |  |  |
| **DAY 3** |  |  |  |  |
| **GENE SYMBOL** | **FC** | **P(Corr)** | **DESCRIPTION** | **KNOWN OR SUSPECTED FUNCTION(S)** |
| **Spink2** | 12.887 | 8.23E-04 | Serine peptidase inhibitor, Kazal type 2 | Modulates apoptotic susceptibility. |
| **Ifng** | 10.57 | 4.05E-04 | IFN-gamma | Immunity (especially intracellular pathogens). |
| **Sv2c** | 6.545 | 1.61E-03 | Synaptic vesicle glycoprotein 2c | Positively regulates vesicle fusion by maintaining the readily releasable pool of secretory vesicles (by similarity). |
| **Cxcl9** | 5.67 | 1.99E-04 | Chemokine (C-X-C motif) ligand 9 | T cell chemoattractant induced by IFN-γ. |
| **Clec4d** | 5.598 | 3.51E-05 | C-type lectin domain family 4, member d (CLECSF8) | Anti-mycobacterial immunity. |
| **Slc44a5** | 5.41 | 2.37E-03 | Solute carrier family 44, member 5 | Caspase-independent cell death. |
| **Cxcl10** | 5.292 | 4.92E-04 | Chemokine (C-X-C motif) ligand 10 | Innate immunity, chemoattractant induced by IFN-γ. |
| **Tnp2** | 5.291 | 1.32E-03 | Nuclear transition protein 2 | Cell cycle. |
| **Adamts4** | 5.248 | 1.76E-04 | A disintegrin-like and metallopeptidase (reprolysin type) with thrombospondin type 1 motif, 4 | Choline transporter-like protein. |
| **Mtfr2** | 5.071 | 1.28E-05 | Mitochondrial fission regulator 2 | Mitochondrial respiration, fission (by similarity). |
| **Rps27a** | 4.881 | 2.21E-03 | Ribosomal protein S27A | Fusion protein consisting of ubiquitin at the N terminus and ribosomal protein S27a at the C terminus. |
| **Il2ra** | 4.793 | 8.24E-04 | IL-2 receptor, alpha chain | T cell differentiation. |
| **Ccl2** | 4.62 | 3.73E-05 | Chemokine (C-C motif) ligand 2 | Recruits monocytes, memory T cells, and DCs. |
| **Zbp1** | 4.534 | 1.70E-03 | Z-DNA-binding protein 1 | Plays a role in the innate immune response by binding to foreign DNA and inducing type-I IFN production. |
| **Bcat1** | 4.478 | 1.01E-03 | Branched chain aminotransferase 1, cytosolic | Metabolism, cell proliferation. |
| **Gbp2** | 4.441 | 9.82E-05 | Guanylate binding protein 2 | IFN-inducible, immunity, intracellular resistance, associates with autophagy protein LC3. |
| **Cth** | 4.401 | 9.17E-04 | Cystathionase (cystathionine gamma-lyase) | Metabolism, produces cysteine. |
| **Bex1** | 4.365 | 8.24E-04 | Brain-expressed gene 1 | Cell cycle. |
| **Dtl** | 4.303 | 6.18E-04 | Denticleless E3 ubiquitin protein ligase homolog (*Drosophila*) | Cell cycle and DNA damage response. |
| **Il12rb1** | 4.23 | 6.55E-05 | IL-12 receptor, beta 1 | Differentiation to Th1, IFN-γ production, enhances activity of NK cells and CD8^+^ T cells. |
| **Plac8** | 4.221 | 2.65E-05 | Placenta-specific 8 | Innate immunity, chromatin binding. |
| **Gm4841** | 4.193 | 2.13E-04 | IFN-inducible GTPase-like (predicted) | Unknown. |
| **Batf2** | 4.185 | 2.55E-07 | Basic leucine zipper transcription factor, ATF-like 2 | AP-1 family transcription factor, differentiation of CD8^+^ DCs. |
| **Igtp** | 4.136 | 8.45E-04 | IFN-γ-induced GTPase | Immune defence. |
| **Slc16a3** | 4.107 | 3.65E-06 | Solute carrier family 16 (monocarboxylic acid transporters), member 3 | Metabolism, negative regulation of cytokines that signal through JAK/STAT3, modulator of IFN-γ action. |
| **Cenpe** | 4.057 | 5.86E-04 | Centromere protein E | Cell cycle, mitosis, MHCI antigen processing and presentation. |
| **Ocstamp** | 3.934 | 1.61E-04 | Osteoclast stimulatory transmembrane protein | Probable cell surface receptor that plays a role in cellular fusion and cell differentiation. |
| **Csf2** | 3.929 | 9.79E-04 | Colony stimulating factor 2 (granulocyte-macrophage) (GM-CSF) | Differentiation of macrophages and granulocytes. |
| **Ect2** | 3.918 | 1.02E-05 | Ect2 oncogene | Cell cycle, mitosis. |
| **Timp1** | 3.863 | 4.57E-04 | Tissue inhibitor of metalloproteinase 1 | Inhibitor of matrix metalloproteases. |
| **Iigp1** | 3.842 | 2.34E-04 | IFN-inducible GTPase 1 | Immunity to intracellular parasites, recruited to the vacuole membrane, destroys the membrane, exposing the parasite. |
| **Sgol2** | 3.84 | 2.19E-04 | Shugoshin-like 2 | Cell cycle, mitosis. |
| **Htr7** | 3.827 | 2.11E-05 | 5-hydroxytryptamine receptor 7 | Serotonin receptor, regulation of circadian rhythms, smooth muscle relaxation, learning and memory. |
| **Hist1h3g** | 3.797 | 3.27E-04 | Histone cluster 1, H3g | DNA replication, transcription. |
| **Tgtp2** | 3.781 | 1.02E-03 | T cell-specific GTPase 2, IFN-γ-inducible GTPase Ifggb5 protein | Unknown. |
| **Ccr4** | 3.735 | 4.47E-04 | Chemokine (C-C motif) receptor 4 | Receptor for chemokines MIP-1, RANTES, TARC and MCP-1. Cell recruitment. |
| **Ncapg** | 3.723 | 1.26E-03 | Non-SMC condensin I complex, subunit G | Cell cycle, mitosis. |
| **Cenph** | 3.684 | 2.88E-05 | Centromere protein H | Cell cycle, mitosis. |
| **Soat2** | 3.661 | 4.84E-06 | Sterol O-acyltransferase 2 | Lipoprotein assembly and dietary cholesterol absorption. |
| **Socs1** | 3.605 | 1.09E-03 | Suppressor of cytokine signaling 1 | Maintains T regulatory cell activity, suppresses IFN-γ and IL-17. |
| **Cenpw** | 3.595 | 1.73E-04 | Centromere protein W | Mitosis. |
| **Dbx2** | 3.547 | 3.15E-04 | Developing brain homeobox 2 | Transcription factor / regulation of transcription. |
| **Trem2** | 3.482 | 1.33E-05 | Triggering receptor expressed on myeloid cells 2 | Functions in immune response and may be involved in chronic inflammation by triggering the production of constitutive inflammatory cytokines. |
| **Cks1brt** | 3.474 | 1.01E-04 | CDC28 protein kinase 1b, retrogene | Cell division, cell cycle. |
| **Npm1** | 3.459 | 3.97E-04 | Nucleophosmin 1 | Cell cycle, mitosis. |
| **Cenpk** | 3.457 | 1.19E-04 | Centromere protein K | Mitosis. |
| **Fignl1** | 3.43 | 3.54E-04 | Fidgetin-like 1 | Involved in DNA double-strand break repair via homologous recombination. |
| **Zbtb32** | 3.423 | 2.29E-04 | Zinc finger and BTB domain-containing 32 | NK cell proliferation, GATA3 inhibition, T helper cell proliferation. |
| **Dnph1** | 3.414 | 2.15E-03 | 2'-deoxynucleoside 5'-phosphate N-hydrolase 1 | Stimulated by c-Myc, a transcription factor involved in cell proliferation, differentiation and apoptosis. |
| **Spc25** | 3.404 | 5.21E-04 | SPC25, NDC80 kinetochore complex component, homolog (*S. cerevisiae*) | May be involved in kinetochore-microtubule interaction and spindle checkpoint activity. |
| **Ly6a** | 3.389 | 2.15E-03 | Lymphocyte antigen 6 complex, locus A | T cell activation, T cell proliferation. |
| **Tacc3** | 3.381 | 2.40E-04 | Transforming, acidic coiled-coil containing protein 3 | Mitosis. |
| **Gbp5** | 3.348 | 3.17E-05 | Guanylate binding protein 5 | IFN-inducible, immunity, intracellular resistance. |
| **Uhrf1** | 3.345 | 1.55E-04 | Ubiquitin-like, containing PHD and RING finger domains, 1. E3 ubiquitin-protein ligase | Protein ubiquitination, acts as a key epigenetic regulator by bridging DNA methylation and chromatin modification. |
| **Irgm2** | 3.335 | 5.82E-04 | Immunity-related GTPase family M member 2 | Immunity, regulation of autophagy, important role in resistance to intracellular pathogens. |
| **Batf** | 3.333 | 5.55E-07 | Basic leucine zipper transcription factor, ATF-like | Transcription factor, mediates the differentiation of Th17, T follicular helper cell, CD8^+^ DCs and class-switch recombination in B cells. |
| **Ly6f** | 3.306 | 3.74E-04 | Lymphocyte antigen 6 complex, locus F | Overexpression increases apoptotic susceptibility. |
| **Serpina3f** | 3.283 | 2.09E-03 | Serine (or cysteine) peptidase inhibitor, clade A, member 3F | Unknown. |
| **Lamc2** | 3.28 | 1.64E-04 | Laminin, gamma 2 | Laminins are involved in cell adhesion, differentiation, migration, signaling, and metastasis. |
| **Gmnn** | 3.275 | 4.87E-04 | Geminin | Cell cycle regulation inhibits DNA replication. |
| **Olfr118** | 3.272 | 9.03E-05 | Olfactory receptor 118 | Detection of chemical stimulus involved in sensory perception of smell, G-protein coupled receptor signaling pathway. |
| **Lmnb1** | 3.27 | 8.13E-05 | Lamin B1 | Lamin proteins are thought to be involved in nuclear stability, chromatin structure and gene expression. |
| **Slc13a3** | 3.242 | 6.88E-05 | Solute carrier family 13 (sodium-dependent dicarboxylate transporter), member 3 | Solute carrier family 13, member 3. |
| **Tcam1** | 3.239 | 1.73E-03 | Testicular cell adhesion molecule 1 | Intercellular adhesion molecule. |
| **Hist1h2ao** | 3.24 | 1.83E-03 | Histone cluster 1, H2ao | Negative regulator of cell proliferation. |
| **Ttll9** | 3.233 | 1.06E-03 | Tubulin tyrosine ligase-like family, member 9 | Probable tubulin polyglutamylase that forms polyglutamate side chains on tubulin. |
| **Cenpi** | 3.195 | 7.94E-03 | Centromere protein I | Cell cycle. |
| **Dut** | 3.135 | 1.62E-04 | Deoxyuridine triphosphatase | Nucleotide metabolism. |
| **Hsp90aa1** | 3.112 | 4.33E-05 | Heat shock protein 90, alpha (cytosolic), class A member 1 | Cell cycle, cell proliferation, Binds LPS and mediates inflammatory response, including TNF secretion by monocytes. |
| **Dnase2b** | 3.107 | 1.21E-03 | Deoxyribonuclease II beta | Hydrolyzes DNA under acidic conditions. |
| **Cdca7** | 3.096 | 6.83E-04 | Cell division cycle-associated 7 | Cell cycle, mitosis, a c-Myc responsive gene. |
| **Hist1h2an** | 3.09 | 4.33E-05 | Histone cluster 1, H2an | Mitosis. |
| **Cdca2** | 3.069 | 1.21E-03 | Cell division cycle-associated 2 | Cell division. |
| **Stmn1** | 3.057 | 6.71E-05 | Stathmin 1 | Caspase independent apoptosis. Proposed to function as an intracellular relay integrating regulatory signals of the cellular environment. |
| **Cks1b** | 3.049 | 1.27E-04 | CDC28 protein kinase 1b | Cell cycle. |
| **Kpna2** | 3.021 | 6.71E-05 | Karyopherin (importin) alpha 2 | Nuclear protein import, adapter protein for nuclear receptor KPNB1. IFN signalling and GPCR pathways. |
| **Ticrr** | 3.02 | 1.27E-04 | TOPBP1-interacting checkpoint and replication regulator | Initiation of DNA replication. |
| **Serpina3g** | 3.012 | 9.10E-04 | Serine (or cysteine) peptidase inhibitor, clade A, member 3G | Promotes the survival of cytotoxic T lymphocytes, allowing them to differentiate into memory CD8^+^ T cells. |
| **Mybl2** | 3 | 1.17E-04 | Myeloblastosis oncogene-like 2 | Transcription factor involved in the regulation of cell survival, proliferation, and differentiation. |
| **Eme1** | 2.99 | 1.61E-05 | Essential meiotic endonuclease 1 homolog 1 (*S. pombe*) | DNA repair, mitosis. |
| **Tmem97** | 2.987 | 1.56E-05 | Transmembrane protein 97 | Control of cellular cholesterol levels. |
| **Timm8a1** | 2.985 | 8.04E-04 | Translocase of inner mitochondrial membrane 8A1 | Import and insertion of some multi-pass transmembrane proteins into the mitochondrial inner membrane. |
| **Knstrn** | 2.965 | 2.22E-04 | Kinetochore-localized astrin/SPAG5 binding | Mitosis. |
| **Cd300lf** | 2.946 | 1.23E-05 | CD300 antigen-like family member F | Regulation of immune response, expressed on myeloid cells. |
| **Ly6i** | 2.919 | 9.67E-05 | Lymphocyte antigen 6 complex, locus I | Induced during early immune response to infection. |
| **Vcan** | 2.913 | 1.46E-03 | Versican | Cell adhesion, proliferation, proliferation, migration and angiogenesis, tissue morphogenesis and maintenance. |
| **Ly6c1** | 2.892 | 2.15E-03 | Lymphocyte antigen 6 complex, locus C1 | Homing of activated cells. |
| **Bard1** | 2.889 | 3.16E-04 | BRCA1 associated RING domain 1 | Cell cycle, DNA damage. |
| **Gvin1** | 2.881 | 9.07E-04 | GTPase, very large IFN-inducible 1 | Unknown. |
| **Rad54l** | 2.881 | 2.15E-04 | RAD54 like (*S. cerevisiae*) | DNA repair and recombination. |
| **Zwilch** | 2.847 | 1.47E-03 | Zwilch kinetochore protein | Cell cycle, mitosis. |
| **Hmgb2** | 2.842 | 2.52E-04 | High mobility group box 2 | DNA binding proteins that associates with chromatin and has the ability to bend DNA. |
| **Gins1** | 2.832 | 3.65E-04 | GINS complex subunit 1 (Psf1 homolog) | DNA replication. |
| **Tubb6** | 2.813 | 2.54E-04 | Tubulin, beta 6 class V | Component of microtubules, mitosis, movement of vesicles and cell organelles. |
| **Cenpm** | 2.807 | 1.81E-03 | Centromere protein M | Cell cycle, mitosis. |
| **Mnda** | 2.807 | 4.16E-04 | Myeloid cell nuclear differentiation antigen | May act as a transcriptional activator/repressor in the myeloid lineage. Plays a role in the granulocyte/monocyte cell-specific response to IFN. |
| **Tvp23a** | 2.806 | 7.02E-05 | Trans-golgi network vesicle protein 23A | Unknown. |
| **Poc1a** | 2.806 | 1.08E-03 | POC1 centriolar protein homolog A (*Chlamydomonas*) | Cell cycle, mitosis. |
| **Car12** | 2.804 | 1.34E-03 | Carbonic anyhydrase 12 | Metabolism. Hypoxia induced, neutralizes acidity. |
| **Uck2** | 2.796 | 1.34E-03 | Uridine-cytidine kinase 2 | Metabolism, DNA and RNA synthesis. |
| **Ndufab1** | 2.794 | 4.69E-05 | NADH dehydrogenase (ubiquinone) 1, alpha/beta subcomplex, 1 | Carrier of the growing fatty acid chain in fatty acid biosynthesis in mitochondria. Subunit of mitochondrial NADH dehydrogenase (Complex I). |
| **Fabp5** | 2.791 | 1.99E-03 | Fatty acid-binding protein 5, epidermal | May play roles in fatty acid uptake, transport, and metabolism. |
| **Hspd1** | 2.79 | 2.00E-04 | Heat shock protein 1 (chaperonin) | May function as a signaling molecule in the innate immune system. |
| **Mcm3** | 2.785 | 2.86E-04 | Minichromosome maintenance-deficient 3 (*S. cerevisiae*) | DNA replication. |
| **Adamts1** | 2.758 | 7.93E-05 | A disintegrin-like and metallopeptidase (reprolysin type) with thrombospondin type 1 motif, 1 | Associated with various inflammatory processes. |
| **Nusap1** | 2.754 | 1.09E-04 | Nucleolar and spindle associated protein 1 | Plays a role in spindle microtubule organization. |
| **Dctpp1** | 2.751 | 3.02E-04 | dCTP pyrophosphatase 1 | Purine metabolism. |
| **Rasl2-9** | 2.744 | 3.46E-04 | MRAS-like, family 2, locus 9 | Cell cycle, mitosis, GTPase. |
| **Tg** | 2.73 | 2.20E-03 | Thyroglobulin | A substrate for the synthesis of thyroxine and triiodothyronine as well as the storage of the inactive forms of thyroid hormone and iodine. |
| **Pask** | 2.722 | 1.22E-05 | PAS domain-containing serine/threonine kinase | Plays a role in insulin expression and adaptation to changing metabolic states. |
| **Icos** | 2.72 | 5.67E-04 | Inducible T cell co-stimulator | Plays an important role in cell-cell signaling, immune responses, and regulation of cell proliferation. |
|  |  |  |  |  |
| **DAY 7** |  |  |  |  |
| **GENE SYMBOL** | **FC** | **p(Corr)** | **DESCRIPTION** | **KNOWN OR SUSPECTED FUNCTION(S)** |
| **Mcpt1** | 4.7488327 | 2.97E-02 | Mast cell protease 1 | Inflammatory mediator. |
| **Grhl3** | 4.2436547 | 5.96E-03 | Grainyhead-like 3 (*Drosophila*) | Epithelial and endothelial cell homeostasis. |
| **Tmem171** | 4.024025 | 1.63E-02 | Transmembrane protein 171 | Unknown. |
| **1810065E05Rik** | 3.474228 | 8.82E-03 | RIKEN cDNA 1810065E05 gene | Unknown. |
| **Gbp7** | 2.902595 | 7.71E-04 | Guanylate-binding protein 7 | IFN-inducible, immunity, intracellular resistance, promote oxidative killing and delivers antimicrobial peptides to autophagolysosomes. |
| **Sfrp4** | 2.8638344 | 4.76E-02 | Secreted frizzled-related protein 4 | Cell growth and differentiation, interacts with Wnt proteins. |
| **Cd300lf** | 2.8186488 | 9.94E-05 | CD300 antigen-like family member F | Acts as an inhibitory receptor for myeloid cells and mast cells. |
| **Entpd3** | 2.744183 | 2.20E-02 | Ectonucleoside triphosphate diphosphohydrolase 3 | Regulation of extracellular levels of ATP and other nucleotides. |
| **Serpina3i** | 2.674789 | 5.09E-03 | Serine (or cysteine) peptidase inhibitor, clade A, member 3I | Inflammatory responses, regulator of proteolysis and lipid metabolic processes. |
| **Clec4d** | 2.6712837 | 8.51E-04 | C-type lectin domain family 4, member d | Anti-mycobacterial immunity, endocytic receptor triggering intracellular signaling, phagocytosis, respiratory burst, cytokine production. |
| **Mmp3** | 2.6600542 | 1.13E-02 | Matrix metallopeptidase 3 | Involved in the breakdown of extracellular matrix in normal physiological processes and disease/healing processes. |
| **Gm9776** | 2.6297865 | 1.07E-02 | Predicted gene 9776 | Unknown. |
| **Gpr55** | 2.5836818 | 2.55E-03 | G protein-coupled receptor 55 | Putative cannabinoid receptor, putative physiological roles include pain signaling, control of vascular tone and inflammation. |
| **Gbp11** | 2.5820422 | 7.15E-04 | Guanylate-binding protein 11 | IFN-inducible, immunity, intracellular resistance. |
| **Gp49a** | 2.571717 | 9.62E-04 | Glycoprotein 49 A | Immunoglobulin superfamily, on mast cell progenitors. |
| **Abca13** | 2.5638509 | 2.08E-02 | ATP-binding cassette, subfamily A | Transmembrane transporter. |
| **Frzb** | 2.5590315 | 4.01E-02 | Frizzled-related protein | Cell growth and differentiation, interact with Wnt proteins. |
| **Gpr31b** | 2.5525725 | 1.09E-02 | cDNA, RIKEN clone:9930022F21:similar to G protein coupled receptor [AK036897] | High-affinity receptor for 12-(S)-HETE, an arachidonic acid metabolite. Ligand-binding leads to activation of ERK1/2 (MAPK3/MAPK1), MEK, and NF-kappa-B. |
| **Fabp3** | 2.528363 | 4.54E-02 | Fatty acid-binding protein 3, muscle and heart | Intracellular transport of long-chain fatty acids and their acyl-CoA esters. |
| **Tnfaip2** | 2.5179718 | 6.51E-03 | Tumor necrosis factor alpha-induced protein 2 | May play a role as a mediator of inflammation and angiogenesis. |
| **Fgl2** | 2.5087934 | 7.74E-04 | Fibrinogen-like protein 2 | May play a role in physiologic lymphocyte functions at mucosal sites. |
| **Gm14446 /Ifit1bl1** | 2.494482 | 1.88E-02 | Predicted gene 14446 /IFN-induced protein with tetratricpeptide repeats 1B like 1 | Unknown. |
| **Jun** | 2.4820018 | 1.20E-02 | Jun proto-oncogene (AP-1, c-Jun) | Transcription factor, PI-3K pathway, MAPK signaling pathway. |
| **Oas1a** | 2.4727151 | 8.42E-03 | 2'-5' oligoadenylate synthetase 1A | Type I IFN-induced, antiviral response, apoptosis, cell growth, differentiation and gene regulation. |
| **Gpr171** | 2.450619 | 1.05E-03 | G protein-coupled receptor 171 | Unknown. |
| **Fam26f** | 2.4386528 | 1.34E-03 | Family with sequence similarity 26, member F | Pore-forming subunit of a voltage-gated ion channel. |
| **Mnda** | 2.4028676 | 4.18E-03 | Myeloid cell nuclear differentiation antigen | May act as a transcriptional activator/repressor in the myeloid lineage. Plays a role in the granulocyte/monocyte cell-specific response to IFN. |
| **Gpr113** | 2.39236 | 1.49E-02 | G protein-coupled receptor 113 | Unknown. |
| **Fcgr1** | 2.3818383 | 4.88E-04 | Fc receptor, IgG, high affinity I | Antibody dependent killing, antigen presentation, inflammatory responses, immune complex formation. |
| **Samhd1** | 2.377107 | 2.57E-03 | SAM domain and HD domain, 1 | May play a role in regulation of the innate immune response. |
| **Il18rap** | 2.3705933 | 9.39E-04 | IL-18 receptor accessory protein | Enhances the IL18-binding activity of the IL18 receptor and plays a role in signaling by IL18. |
| **Gvin1** | 2.3642375 | 2.40E-04 | GTPase, very large IFN-inducible 1 | Unknown. |
| **Phf11b** | 2.359645 | 5.13E-03 | PHD finger protein 11B | Positive regulator of Th1-type cytokine gene expression. |
| **Lilrb4** | 2.3591342 | 2.49E-03 | Leukocyte immunoglobulin-like receptor, subfamily B, member 4 | Binds to MHC class I molecules on antigen-presenting cells and downregulates immune responses. Can also function in antigen capture and presentation. |
| **Clec4a1** | 2.358264 | 1.03E-04 | C-type lectin domain family 4, member a1 (DC inhibitory receptor 4) | Pattern recognition receptor. |
| **Tmevpg1** | 2.3180237 | 2.48E-02 | Theiler's murine encephalomyelitis virus persistence candidate gene 1 | Associated with upregulated IFN-γ. |
| **Oas1f** | 2.3156147 | 3.61E-03 | 2'-5' oligoadenylate synthetase 1F | Type I IFN-induced, antiviral response, apoptosis, cell growth, differentiation and gene regulation. |
| **Gbp3** | 2.307105 | 5.22E-03 | Guanylate-binding protein 3 | IFN-inducible, immunity, intracellular resistance. |
| **Sytl3** | 2.3053212 | 4.34E-02 | Synaptotagmin-like 3 | May play a role in vesicle trafficking. |
| **Hdc** | 2.3039324 | 2.43E-02 | Histidine decarboxylase | Catalyzes the biosynthesis of histamine from histidine. |
| **BC068157 (Prr36)** | 2.291812 | 2.04E-02 | cDNA sequence BC068157/proline rich 36 | Unknown. |
| **Pa2g4** | 2.2889938 | 2.72E-02 | Proliferation-associated 2G4 | Regulates cell proliferation, differentiation, and survival. |
| **Gatm** | 2.2853074 | 2.64E-04 | Glycine amidinotransferase (L-arginine:glycine amidinotransferase) | Catalyzes the biosynthesis of guanidinoacetate, the immediate precursor of creatine. |
| **Gm4610** | 2.2792337 | 2.36E-03 | Predicted gene 4610 | Unknown. |
| **Pnp** | 2.266963 | 9.62E-04 | Purine-nucleoside phosphorylase | Reversibly catalyzes the phosphorolysis of purine nucleosides. Mutations affect T and B cell immunity. |
| **Gbp8** | 2.265515 | 3.49E-03 | Guanylate-binding protein 8 | IFN-inducible, immunity, intracellular resistance. |
| **Rundc3b** | 2.2446773 | 2.91E-02 | RUN domain-containing 3B | Unknown. |
| **C430042M11Rik** | 2.2347004 | 2.34E-03 | RIKEN cDNA C430042M11 gene | Unknown. |
| **Rbm3os** | 2.1765015 | 2.37E-02 | RNA-binding motif protein 3, opposite strand | Unknown. |
| **Dtx3l** | 2.1654627 | 2.69E-03 | Deltex 3-like (*Drosophila*) | Ubiquitin ligase that mediates monoubiquitination of Lys-91 of histone H4 in response to DNA damage. |
| **Trpm6** | 2.1435318 | 4.13E-03 | Transient receptor potential cation channel, subfamily M, member 6 | Essential ion channel and serine/threonine-protein kinase. Crucial for magnesium homeostasis. |
| **Upp1** | 2.1415446 | 1.48E-02 | Uridine phosphorylase 1 | Catalyzes the reversible phosphorylytic cleavage of uridine and deoxyuridine to uracil and ribose- or deoxyribose-1-phosphate. |
| **2210011C24Rik** | 2.13619 | 3.92E-02 | RIKEN cDNA 2210011C24 gene | Unknown. |
| **Ddx21** | 2.136163 | 6.09E-04 | DEAD (Asp-Glu-Ala-Asp) box polypeptide 21 | RNA helicase that promotes rRNA transcription, processing and modification. |
| **Phf11a** | 2.1322963 | 5.21E-03 | PHD finger protein 11A | Positive regulator of Th1-type cytokine gene expression. |
| **Ropn1l** | 2.1252851 | 2.59E-04 | Ropporin 1-like | Involved in respiratory cilia motility and sperm motility. |
| **5730508B09Rik** | 2.125156 | 1.41E-03 | RIKEN cDNA 5730508B09 gene | Unknown. |
| **Fbln5** | 2.116185 | 3.74E-02 | Fibulin 5 | Essential for elastic fiber formation, is involved in the assembly of continuous elastin polymer and promotes the interaction of microfibrils and elastin. |
| **Gpr82** | 2.1126857 | 2.44E-03 | G protein-coupled receptor 82 | Unknown. |
| **Gm1966** | 2.1101246 | 1.68E-03 | Predicted gene 1966 | Unknown, possible pseudogene. |
| **Ccl4** | 2.1059403 | 1.72E-02 | Chemokine (C-C motif) ligand 4 | Monokine with inflammatory and chemokinetic properties. Binds to CCR5. |
| **Hat1** | 2.1012108 | 3.79E-03 | Histone aminotransferase 1 | May be involved in nucleosome assembly during DNA replication and repair. |
| **Parp9** | 2.0948584 | 3.21E-03 | Poly (ADP-ribose) polymerase family, member 9 | Involved in inducing the expression of IFN-gamma-responsive genes, plays a role in PARP1-dependent DNA damage repair. |
| **Pml** | 2.0886188 | 2.43E-02 | Promyelocytic leukemia | Functions in important cellular processes including tumor suppression, transcriptional regulation, apoptosis, senescence, DNA damage response, and viral defence mechanisms. |
| **Fasl** | 2.08548 | 1.89E-02 | Fas ligand (TNF superfamily, member 6) | Induction of apoptosis triggered by binding to FAS, essential for immune system regulation, including activation-induced cell death of T cells and Cytotoxic T lymphocyte induced cell death. |
| **Gm9519** | 2.084688 | 2.61E-02 | Predicted gene 9519 | Unknown. |
| **Spon1** | 2.0806322 | 5.26E-03 | Spondin 1 (f-spondin) extracellular matrix protein | Cell adhesion protein. |
| **Tma16** | 2.0754251 | 1.30E-03 | Translation machinery associated 16 homolog (*S. cerevisiae*) | Unknown. |
| **Vill** | 2.0746076 | 1.82E-03 | Villin-like (Vill), transcript variant 1, mRNA | Possible tumor suppressor. May play a role in actin bundling. |
| **AW011738** | 2.0733292 | 2.83E-02 | Expressed sequence AW011738 | Unknown. |
| **Ackr2** | 2.0689807 | 3.97E-03 | Atypical chemokine receptor 2 (CCR10) | Scavenges chemokines. Acts as a regulator of inflammatory leukocyte interactions with lymphatic endothelial cells and is required for immature/mature DC discrimination. Plays a major role in the immune silencing of macrophages during the resolution of inflammation. |
| **Gdap10** | 2.0682821 | 6.65E-03 | Ganglioside-induced differentiation-associated protein 10 | Unknown. |
| **Pfkp** | 2.0575264 | 1.48E-04 | phosphofructokinase, platelet | Catalyzes the phosphorylation of D-fructose 6-phosphate to fructose 1,6-bisphosphate by ATP, the first committing step of glycolysis. |
| **Crem** | 2.0558362 | 4.17E-03 | cAMP responsive element modulator | An important component of cAMP-mediated signal transduction. |
| **Ms4a4c** | 2.054933 | 1.56E-03 | Membrane-spanning 4-domains, subfamily A, member 4C | May be involved in signal transduction as a component of a multimeric receptor complex. |
| **Pim2** | 2.0535476 | 1.06E-03 | Proviral integration site 2 | Involved in cell survival and cell proliferation. |
| **Rasgef1c** | 2.050515 | 3.78E-02 | RasGEF domain family, member 1C | Guanine nucleotide exchange factor. |
| **1500012F01Rik** | 2.050338 | 2.41E-03 | RIKEN cDNA 1500012F01 gene | Unknown. |
| **Cebpb** | 2.0487304 | 2.09E-04 | CCAAT/enhancer binding protein beta | Important transcription factor regulating the expression of genes involved in immune and inflammatory responses. |
| **Dnajc5** | 2.0432081 | 2.67E-02 | DnaJ (Hsp40) homolog, subfamily C, member 5 | Plays a role in membrane trafficking and protein folding, and has been shown to have anti-neurodegenerative properties. |
| **Wfdc17** | 2.0426126 | 5.07E-03 | WAP four-disulfide core domain 17 | Unknown. WAP domain has antimicrobial and immune regulatory activity. |
| **Gbp9** | 2.0367005 | 1.65E-02 | Guanylate-binding protein 9 | IFN-inducible, immunity, intracellular resistance. |
| **Samhd1** | 2.0335665 | 7.48E-04 | SAM domain and HD domain, 1 | May play a role in mediating proinflammatory responses to TNF-α signaling. |
| **Rtp4** | 2.0299013 | 4.33E-02 | Receptor transporter protein 4 | Unknown. IFN-induced. |
| **St14** | 2.0285437 | 9.96E-04 | Suppression of tumorigenicity 14 | An epithelial-derived, integral membrane serine protease. Degrades extracellular matrix. |
| **Brsk2** | 2.0204654 | 2.19E-02 | BR serine/threonine kinase 2 | Plays a role regulation of the mitotic cell cycle, insulin secretion and neuron polarization. May play a role in the apoptotic response triggered by ER stress. |
| **Vcan** | 2.016935 | 8.21E-03 | Versican | May play a role in intercellular signaling and in connecting cells with the extracellular matrix. May take part in the regulation of cell motility, growth and differentiation. |
| **Il12b** | 2.0128756 | 5.23E-03 | Interleukin-12B | Immunity, component of IL-12 and IL-23. |
| **Ndc80** | 2.012406 | 6.80E-03 | NDC80 homolog, kinetochore complex component (*S. cerevisiae*) | Acts as a component of the essential kinetochore-associated NDC80 complex, which is required for chromosome segregation and spindle checkpoint activity. |
| **Cybb** | 2.0099216 | 2.14E-03 | Cytochrome b-245, beta polypeptide | Bacterial killing, critical component of the membrane-bound oxidase of phagocytes that generates superoxide. |
| **Bcl2a1c** | 2.008379 | 2.40E-04 | B cell leukemia/lymphoma 2-related protein A1c | Reduces the release of pro-apoptotic cytochrome c from mitochondria and block caspase activation. |
| **Nudt21** | 2.00786 | 2.25E-04 | Nudix (nucleoside diphosphate-linked moiety X)-type motif 21 | 3' RNA cleavage and polyadenylation processing. |
| **LOC102634581** | 2.00643 | 6.11E-04 | Predicted: nuclear body protein SP140-like | Unknown. |
| **Ctsc** | 2.0064008 | 3.11E-04 | Cathepsin C | A lysosomal cysteine proteinase that appears to be a central coordinator for activation of many serine proteinases in immune/inflammatory cells. |
| **Lss** | 2.0062501 | 2.60E-03 | Lanosterol synthase | Catalyzes the cyclization of (S)-2,3 oxidosqualene to lanosterol, a reaction that forms the sterol nucleus. |
| **Stat1** | 2.0041971 | 3.76E-03 | Signal transducer and activator of transcription 1 | Mediates cellular responses to IFNs, cytokine KITLG/SCF and other cytokines and other growth factors. |
| **Cacybp** | 2.0024102 | 5.84E-04 | Calcyclin-binding protein | May be involved in calcium-dependent ubiquitination and subsequent proteasomal degradation of target proteins. |
| **Sod2** | 2.000827 | 6.84E-04 | Superoxide dismutase 2, mitochondrial | Destroys superoxide anion radicals which are normally produced within the cells and which are toxic to biological systems. |
| **Ctla4** | 2.0004318 | 1.71E-04 | Cytotoxic T-lymphocyte-associated protein 4 | Inhibitory receptor acting as a major negative regulator of T-cell responses. |

^a^ Venn diagrams were plotted in GeneSpring to analyze overlap of genes significantly upregulated (*p*<0.05) at least 2-fold compared to naïve control in groups vaccinated with different strains of BCG, at 1, 3 and 7 days post-vaccination. Lists of genes upregulated specifically in BCG Δ*ureC*::*hly* Δ*nuoG* mice are given here with their potential or confirmed protein functions. Non-coding genes and confirmed pseudogenes were not included.

^b^ The *p* values corrected for multiple comparisons are shown.

^c^ Gene/protein functions were obtained from Mouse Gene Detail (MGI) (http://www.informatics.jax.org), GeneCards (http://www.genecards.org/), National Centre for Biotechnology information (NCBI) (http://www.ncbi.nlm.nih.gov/) and Uniprot (http://www.uniprot.org) online repositories.
