# Supplementary material for: Deletion of nuoG from the Vaccine Candidate Mycobacterium bovis BCG ΔureC::hly Improves Protection against Tuberculosis
Source: mBio. 2016 May 24;7(3):e00679-16. doi: 10.1128/mBio.00679-16 (PMC4895111; doi:10.1128/mBio.00679-16)
Supplement: Table S3 — GO enrichment analysis of genes identified as significantly upregulated (P < 0.05) at least 2-fold compared to naive controls only in BCG ΔureC::hly ΔnuoG-vaccinated mice. GeneSpring was used to perform GO analysis on genes significantly upregulated compared to naive controls in BCG ΔureC::hly ΔnuoG mice only, as identified by Venn diagrams. At day 1, GO terms indicated involvement of genes involved in acute inflammation, while at day 3 they indicated immune activation and host defense processes as well as cell proliferation and differentiation. By day 7, developmental and cell cycle processes dominated the list of GO terms in the enlarged lymph node, and so only the first 20 hits are included in the list. The P values corrected for multiple comparisons are shown. [file mbo003162827st3.doc]

**Table S3** GO enrichment analysis of genes identified as significantly upregulated (*p*<0.05) at least 2-fold compared to naïve controls only in BCG Δ*ureC*::*hly* Δ*nuoG-*vaccinated mice.^a^

| **GO term** | **P value** | **Adjusted P value for multiple comparisons** |
| --- | --- | --- |
| **DAY 1** |  |  |
| Acute inflammatory response | 4.99E-10 | 3.66E-06 |
| Chemotaxis | 2.80E-09 | 1.14E-05 |
| Taxis | 2.95E-09 | 1.14E-05 |
| Acute phase response | 3.36E-09 | 1.14E-05 |
| Cell chemotaxis | 8.69E-09 | 2.13E-05 |
| Response to external stimulus | 1.61E-07 | 3.38E-04 |
| Inflammatory response | 3.27E-07 | 6.41E-04 |
|  |  |  |
| **DAY 3** |  |  |
| Cell cycle | 1.06E-10 | 9.99E-07 |
| Cell cycle process | 7.85E-10 | 4.95E-06 |
| Defense response to protozoan | 1.36E-09 | 6.42E-06 |
| Response to protozoan | 3.18E-09 | 8.94E-06 |
| Immune response | 8.43E-09 | 1.99E-05 |
| Response to cytokine | 2.32E-08 | 4.40E-05 |
| Response to stress | 3.63E-08 | 6.24E-05 |
| Cell division | 5.05E-08 | 7.96E-05 |
| Immune system process | 5.59E-08 | 8.13E-05 |
| Organelle fission | 3.41E-07 | 4.61E-04 |
| Defence response | 4.12E-07 | 5.09E-04 |
| Mitotic cycle | 4.31E-07 | 5.09E-04 |
| Regulation of cell cycle | 8.28E-07 | 8.70E-04 |
| Cellular response to cytokine synthesis | 1.23E-06 | 1.00E-03 |
| DNA metabolic process | 1.30E-06 | 1.00E-03 |
| Nuclear division | 1.37E-06 | 1.00E-03 |
| Mitosis | 1.37E-06 | 1.00E-03 |
| Defense response to other organism | 1.85E-06 | 2.00E-03 |
| Response to IFN-γ | 4.43E-06 | 3.00E-03 |
| Chromosome segregation | 5.20E-06 | 4.00E-03 |
| Single-organism cellular process | 6.91E-06 | 5.00E-03 |
| Cellular response to IFN-γ | 8.72E-06 | 6.00E-03 |
| Response to other organism | 2.68E-05 | 1.10E-02 |
| Immune system development | 2.96E-05 | 1.20E-02 |
| Innate immune response | 3.37E-05 | 1.30E-02 |
| Single organism process | 3.46E-05 | 1.30E-02 |
| Positive regulation of T cell activation | 3.61E-05 | 1.40E-02 |
| Myeloid leukocyte differentiation | 3.79E-05 | 1.40E-02 |
| Response to biotic stimulus | 4.30E-05 | 1.50E-02 |
| Myeloid DC differentiation | 5.07E-05 | 1.70E-02 |
| GTP catabolic process | 5.93E-05 | 2.00E-02 |
| Guanosine-containing compound catabolic process | 6.78E-05 | 2.20E-02 |
| GTP metabolic process | 8.80E-05 | 2.80E-02 |
| Regulation of cell cycle process | 1.05E-04 | 3.20E-02 |
| Myeloid DC activation | 1.12E-04 | 3.40E-02 |
| Guanosine containing compound metabolic process | 1.16E-04 | 3.40E-02 |
| Response to organic substance | 1.20E-04 | 3.50E-02 |
| Single organism organelle organisation | 1.42E-04 | 4.00E-02 |
| Cellular response to DNA damage stimulus | 1.71E-04 | 4.50E-02 |
| Regulation of microtubule based process | 1.77E-04 | 4.50E-02 |
| Regulation of chromosome segregation | 1.80E-04 | 4.50E-02 |
| Cellular response to IFN-β | 1.80E-04 | 4.50E-02 |
| Dendritic cell differentiation | 1.80E-04 | 4.50E-02 |
| Myeloid cell differentiation | 1.93E-04 | 4.60E-02 |
| Microtubule cytoskeleton organisation | 2.00E-04 | 4.70E-02 |
|  |  |  |
| **DAY 7 (top 20)^b^** |  |  |
| System development | 1.10E-25 | 3.23E-21 |
| Multicellular organismal development | 2.49E-25 | 5.49E-21 |
| Cell cycle | 9.14E-25 | 1.61E-20 |
| Developmental process | 1.61E-23 | 2.36E-19 |
| Single organism development process | 2.27E-23 | 2.85E-19 |
| Anatomical structure development | 1.81E-22 | 1.99E-18 |
| Cell cycle process | 1.03E-21 | 9.08E-19 |
| Nuclear division | 2.83E-21 | 2.26E-17 |
| Single organism process | 4.59E-21 | 3.11E-17 |
| Mitotic cell cycle | 3.05E-20 | 1.79E-16 |
| Organelle fusion | 1.49E-19 | 1.14E-16 |
| Biological adhesion | 3.55E-19 | 1.73E-15 |
| Mitosis | 9.76E-19 | 4.52E-15 |
| Cell division | 1.44E-18 | 6.36E-15 |
| Positive regulation of cellular process | 1.13E-17 | 4.72E-14 |
| Single organism cellular process | 1.92E-17 | 7.68E-14 |
| Organ development | 2.68E-17 | 9.82E-14 |
| Cell adhesion | 3.15E-17 | 1.11E-13 |
| Regulation of cell proliferation | 2.29E-16 | 7.74E-13 |
| DNA replication | 2.91E-16 | 9.48E-13 |

^a^ GeneSpring was used to perform GO analysis on genes significantly upregulated compared to naïve controls in BCG Δ*ureC*::*hly* Δ*nuoG* mice only, as identified by Venn diagrams.

^b^ By day 7, developmental and cell cycle processes dominated the list of GO terms in the enlarged lymph node, and so only the first 20 hits are included in the list.
