# Supplementary material for: Deletion of nuoG from the Vaccine Candidate Mycobacterium bovis BCG ΔureC::hly Improves Protection against Tuberculosis
Source: mBio. 2016 May 24;7(3):e00679-16. doi: 10.1128/mBio.00679-16 (PMC4895111; doi:10.1128/mBio.00679-16)
Supplement: Table S4 — Primers for RT-PCR. [file mbo003162827st4.docx]

**Table S4.** Primers for RT-PCR.

| **Gene** | **Forward primer** | **Reverse primer** |
| --- | --- | --- |
| Ywhaz | AGCAGGCAGAGCGATATGAT | TTCTCAGCACCTTCCGTCTT |
| IL1b | GCCCATCCTCTGTGACTCAT | AGGCCACAGGTATTTTGTCG |
| IL18 | GCCTCAAACCTTCCAAATCA | TGGATCCATTTCCTCAAAGG |
| Ifi204 | ATTCTGGATTGGGCAAACTG | CTCTTCCTGGGTTGCAGAAG |
| Gbp2 | ACCAGCTGCACTATGTGACG | TCAGAAGTGACGGGTTTTCC |
| Gbp3 | GTCTGGAGAACGCAGTGACA | GTGCTCCATGAAGACAGCAA |
| Gbp5 | AAAGGCCATTGGTCACTACG | AAGCATCCGCGTTCTTCTTA |
